# Plant-Based Assessment of Inherent Soil Productivity and Contributions to China’s Cereal Crop Yield Increase since 1980

**DOI:** 10.1371/journal.pone.0074617

**Published:** 2013-09-18

**Authors:** Mingsheng Fan, Rattan Lal, Jian Cao, Lei Qiao, Yansen Su, Rongfeng Jiang, Fusuo Zhang

**Affiliations:** 1 Center for Resources, Environment and Food Security, China Agricultural University, Beijing, P. R. China; 2 Carbon Management and Sequestration Center, the Ohio State University, Columbus, Ohio, United States of America; International Rice Research Institute, Philippines

## Abstract

**Objective:**

China’s food production has increased 6-fold during the past half-century, thanks to increased yields resulting from the management intensification, accomplished through greater inputs of fertilizer, water, new crop strains, and other Green Revolution’s technologies. Yet, changes in underlying quality of soils and their effects on yield increase remain to be determined. Here, we provide a first attempt to quantify historical changes in inherent soil productivity and their contributions to the increase in yield.

**Methods:**

The assessment was conducted based on data-set derived from 7410 on-farm trials, 8 long-term experiments and an inventory of soil organic matter concentrations of arable land.

**Results:**

Results show that even without organic and inorganic fertilizer addition crop yield from on-farm trials conducted in the 2000s was significantly higher compared with those in the 1980s — the increase ranged from 0.73 to 1.76 Mg/ha for China’s major irrigated cereal-based cropping systems. The increase in on-farm yield in control plot since 1980s was due primarily to the enhancement of soil-related factors, and reflected inherent soil productivity improvement. The latter led to higher and stable yield with adoption of improved management practices, and contributed 43% to the increase in yield for wheat and 22% for maize in the north China, and, 31%, 35% and 22% for early and late rice in south China and for single rice crop in the Yangtze River Basin since 1980.

**Conclusions:**

Thus, without an improvement in inherent soil productivity, the ‘Agricultural Miracle in China’ would not have happened. A comprehensive strategy of inherent soil productivity improvement in China, accomplished through combining engineering-based measures with biological-approaches, may be an important lesson for the developing world. We propose that advancing food security in 21st century for both China and other parts of world will depend on continuously improving inherent soil productivity.

## Introduction

China, the world’s most populous country of 1.3 billion with a rapidly growing economy, faces a major challenge of achieving food security [1]. Food demand in China also impacts global food supply [2], and the worldwide use of natural resources. China’s agricultural production has witnessed a marked growth in the past half-century, creating the “Agricultural Miracle in China,” which feeds 20% of the world’s population on merely 7% of the global arable land while using only 5% of the planet’s renewable fresh water resources [1,3]. Increase in agricultural output is mainly attributed to the increased yield per unit area of land already under agriculture, resulting from the “Green Revolution” using modern high-yielding varieties. In addition, high yields also have been realized from intensive use of chemical fertilizers, irrigation, and weed and pest control [1,4].

There is a considerable research interest in quantifying the effects of multifactor global change including N application, N deposition, climate change and tropospheric ozone (O_3_) on China’s crop productivity [5,6,7]. Yet, few if any, studies exist to assess the impacts of changes in the underlying quality of soils under on-farm conditions on crop productivity growth in China. Globally, because of the lack of comprehensive data linking soil resource to agricultural productivity, the projections of future crop production do not include changes in soil resource base as a determinant of productivity, nor the building of soil capital and other land improvements as critical components of agricultural investment [8,9,10].

Soil productivity is a result of how well the soil is able to receive and store water and nutrients and create favorable environment for all plant root growth and functions [11]. For the past century, however, nothing has mattered more for soils than their relations with human communities [12], so that inter-temporal degradation of agricultural soil resources are associated worldwide with the inappropriate use of agricultural practices, even under favorable production environments [8]. In developing world such as in south Asia [13-16] and in tropical Africa [17], losses of soil organic matter (SOM) and the attendant decline in soil productivity constitute a major problem that is getting worse. Agricultural soils in China were also vulnerable to degradation processes such as acidification due to overuse of N fertilizer [18], accelerated erosion [19], and secondary salinization [20]. However, conversion to an appropriate agricultural land use and management has been one of major investments in achieving food security for China since the 1950s [3], as designed to increase the quantity and quality of cultivated land across the country. Therefore, what is available evidence which indicates that changes in the underlying quality of soils has effected on crop productivity growth in China? Understanding the effects of past trends can help to gauge how innovative interventions may improve crop yield and enhance environment in the future.

The results presented here are based on the analysis of three unique national data sets: (1) SOM and yields in plots without fertilizer and under best management practices (BMPs) for 1-2 year on-farm trials conducted during 1980s and 2000s respectively, (2) yields in fertilizer omission plots in typical long-term experiments (LTEs) across major irrigated cereal-based cropping systems in China, and (3) an inventory of SOM concentrations of arable land. These data sets were used to test two hypothesis: (1) changes in yield in control plots in on-farm trials reflect changes in the inherent soil productivity in China’s major irrigated cereal-based cropping systems; and (2) increase in yields for major cereal crops and the ‘Agricultural Miracle in China’ would not have happened without improvement in inherent soil productivity. Here, inherent soil productivity is defined as an inherent capacity of the soil to perform productive functions and provide numerous ecosystem services.

## Materials and Methods

### Study Areas and cropping systems

Wheat (*Triticum aestivum* L.), maize (*Zea mays*.L) and rice (*Oryza sativa* L.) are the principal food staples in China. Temporal change in inherent soil productivity and its significance to crop yield growth were studied for five cereal-based cropping systems practiced in different regions. These cropping systems were: (1) winter wheat in north China; (2) summer maize in north China; (3) early rice crop in south China; (4) late rice crop in south China; and (5) single rice crop in Yangtze River Basin. These systems represent irrigated and higher productivity cropping systems. In total, they account for more than 50% of total production of wheat, 40% of maize, and 85% of rice. Overview of the cropping systems, geographical location and provinces involved in the study are shown in Table 1.

**Table 1:** Overview of cropping systems, geographical location and provinces involved in the study.

Number	Regions	Cropping systems	Geographical location	Provinces (including municipalities)
(1)	North	Winter wheat	32-41 °N, 113-120 °E	Beijing, Tianjin, Hebei, Shanxi, Henan, Shandong
(2)		Summer maize		
(3)	South	Early rice	18-26 °N, 110-116 °E	Hubei, Hunan, Jiangxi, Guangxi, Guangdong, Fujian
(4)		Late rice		
(5)	Yangtze River Basin	Single rice	30-31°N,117-121°E	Jiangsu, Anhui, Zhejiang, Chongqing, Sichuan, Hubei, Guizhou

### Plant based assessment of inherent soil productivity

Based on the unique and national scale data sets, inherent soil productivity under on-farm conditions were assessed by using a plant-based agronomic approach. In this approach, inherent soil productivity was used to refer to the actual yield of usable vegetation without nutrient-input addition. Thus, inherent soil productivity was estimated by assessing grain yields in plots without added fertilizer (control plots). These control plots received neither organic amendments (i.e., manure and crop residues) nor chemical fertilizers (e.g., N,P,K) during 1-2 years experimental period. However, control plots received weed, insect and disease control measures, and also supplemental irrigation. During the non-assessment period, the fields received normal farmer’s management practices including organic amendments and/or chemical fertilizers application and other management measures.

Historical trends in inherent soil productivity were established by comparing two data sets of yields from plots without added fertilizer (1980s and 2000s). The results from plant-based approach are verified by checking the relationships between yield in control plots and under BMPs (e.g., N,P,K chemical fertilizers application) and SOM concentration, and historical trends in yield in control plots in LTEs. The control plots in LTEs did not receive any inorganic and organic fertilizer since the time when experiments were established, but received other BMPs such as weed, insect and disease control measures and irrigation, and also regularly adopted the newly released high-yielding varieties.

### Data set collected

For the five cereal-based cropping systems, the crop yield data were collected from on-farm experiments conducted for 1-2 years during 1980-1989 (1980s) and 2000-2010 (2000s). Each of these experiments included a control plot without fertilizer addition and others that received recommended rates of fertilizers. One of these fertilizer plots (e.g., the one with N,P,K chemical fertilizer application but with similar other management practices as used in the control plots) was identified as the treatment with BMPs. Yields of crops without added fertilizer (Yield-CK) and BMPs (Yield-BMPs) were obtained from these experiments. Most of data involved in the first assessment (the 1980s) were from experiments of National Chemical Fertilizer Test Network. Most of the data for 2000s were obtained from on-farm experiments conducted under the auspices of National Soil Test and Fertilizer Recommendation projects (2006-2009). These data are representative of the principal irrigated cereal- based cropping systems, and reflect on-farm conditions in both 1980s and 2000s.

Of the total of 7410 sites, 621 were for wheat (n=271 in 1980s and n=350 in 2000s), 457 for maize (n=200 in the 1980s and n=257 in the 2000s), and 6332 for rice (n=115 for single rice, n=307 for early rice and n=236 for late rice in the 1980s; and n=3011 for single rice, n=1307 for early rice and n=1356 for late rice in the 2000s). The literatures and documents from which the data were derived were listed in supporting online materials as supporting references [References S1-S151 in File S1]. The geographical distribution of the sites is shown in supporting materials (Figure S1). We further summarized 4638 sites (wheat, n= 354; maize, n=425; early rice, n=697; late rice, n=688; single rice, n=2474), which recorded SOM concentration as a basic soil propriety, to form a subset of the data. The sub-data set was used to test any relations between cereal yield (under BMPs and in control plots) and SOM concentration.

To objectively evaluate the changes in SOM between 1980s and 2000s in different cropping systems, an inventory of SOM concentrations from 68 articles [Reference S152-S220 in File S1] were summered (Table S1 in File S1).

Data of crop yields and SOM concentration were collected on plots without fertilizer (without NPK fertilizer application and organic amendments for 13 to 27 years) from typical LTEs established in 1980s or 1990s, which included 3 experiments on wheat-maize in north China, 2 on single rice in Yangtze River Basin and 3 on double rice in south China, and comprised the major LTEs in China’s irrigated cereal-based cropping systems. In these LTEs, yield data from control plots were obtained either annually or every 3 or 5 years as indicative of any temporal changes in agronomic productivity. The data-derived from literature and other documents are listed in supporting online reference materials [Reference S221-S226 in File S1].

### Data treatment and statistics

Paired yield data from control plots and under BMPs in on-farm trials for all five cereal-based cropping systems were grouped according to the Yield-CK with increment of 2 Mg/ha over 1980s and 2000s. The relationship between SOM concentration and Yield-CK and Yield-BMPs were generalized within each group. Further, the relative contribution (RCi%) of inherent soil productivity to Yield-BMPs and the stability index (SI_i_) [21], of yield under BMPs were evaluated by using the following equations 1 and 2 within each group.

$$RC_{i}(\%)=\frac{Yield\text{-}CKi}{Yield\text{-}BMPsi}*100$$ (Eq 1)

Where, Yield-CKi and Yield-BMPsi are the mean yields in control plots and under BMPs of each group (i).

$$SI_{i}(\%)=\frac{STD\left(Yield\text{-}BMPsi\right)}{Yield\text{-}BMPsi}*100$$ (Eq 2)

Where, standard deviation of Yield-BMPsi (*STD (Yield-BMPsi*)) and Yield-BMPsi are yield variation and mean yield under BMPs of each group (i).

Increase in crop yield(△Yield) since 1980, defined as the difference between Yield-CK in the 1980s and Yield-BMPs in 2000s, and the contributions of inherent soil productivity improvement (CI%) to yield increase were quantified by using the equations 3 and 4. The average values from the data set for Yield-CK and Yield-BMPs for each component of equations 3 and 4 are used for the estimations.

ΔYield=Yield-BMPs−Yield-CK (Eq 3)

Where, Yield-BMPs represent yields under BMPs in 2000s, and Yield-CK represent those in control plots in 1980s.

$$CI(\%)=\frac{\Delta Yield\text{-}CK}{\Delta Yield}*100$$ (Eq 4)

Where, △Yield-CK represent the change in yield in control plots between 1980s and 2000s. △Yield is difference between Yield-CK in 1980s and Yield-BMPs in 2000s (See Eq 3).

Yield dynamics over time during the study periods in plots without fertilizer were summarized for each LTEs. The Yield-CK in on-farm trials from the five cropping systems in 1980s were compared with those in 2000s. An unpaired t-test was conducted for statistical comparison of Yield-CK to assess the significance of differences between 1980s and 2000s. Linear regressions between yield-CK and Yield-BMPs for all five cropping systems over 1980s and 2000s were generalized by using 7410 paired data from on-farm trials. Factors tested were considered to be statistically significant at p < 0.05. All statistical analyses were performed using SAS (SAS Institute, 2004).

## Results

### The historical trends in yield in without fertilization plots on-farm in major irrigated cereal-based cropping systems

The data set involving Yield-CK from 7410 on-farm trials done for 1-2 years during 1980s and 2000s period were examined. Despite some differences in the magnitude of the increase among cropping systems, ranging from 0.73 Mg/ha to 1.76 Mg/ha, a significant increase in crop yields occurred between 1980s and 2000s (Table 2). The increase in Yield-CK during this period was 60% for wheat, 15% for maize, and 31%, 37% and 16%, respectively, for rice -based systems (early and late rice in south China and single rice in Yangtze River Basin).

**Table 2:** Changes in China’s wheat, maize, and rice grain yields in control plots without fertilizer addition under on-farm trials (Yield-CK) for major irrigated cereal-based cropping systems between 1980s and 2000s.

		Yield-CK^†^ (Mg/ha)		
Region	Cropping system	1980s	2000s	Increase
North	Winter wheat	2.91 (±1.14)	4.67 (±1.29)	1.76**
	Summer maize	4.91 (±1.44)	5.64 (±1.40)	0.73**
South	Early rice	3.45 (±0.86)	4.54 (±1.01)	1.08**
	Late rice	3.39 (±1.06)	4.66 (±1.07)	1.27**
Yangtze River Basin	Single rice	4.80 (±1.00)	5.60 (±1.14)	0.79**

Note: ** p<0.01; data were showed as average (±std deviation);

† Yield data in control plots were collected from on-farm trials conducted for 1-2 years during 1980s and 2000s periods, respectively. Of the total of 7410 sites, 621 were for wheat (n=271 in 1980s and n=350 in 2000s), 457 for maize (n=200 in the 1980s and n=257 in the 2000s), and 6332 for rice (n=307 for early rice, n=236 for late rice and n=115 for single rice in the 1980s; and n=1307 for early rice, n=1356 for late rice and n=3011 for single rice in the 2000s). The literatures and documents data-derived are listed in supporting online materials as supporting references [Reference S1-S151 in File S1).

### The evidences of yield trends in control plots on-farm reflect mainly change in inherent soil productivity

**Figure 1:**
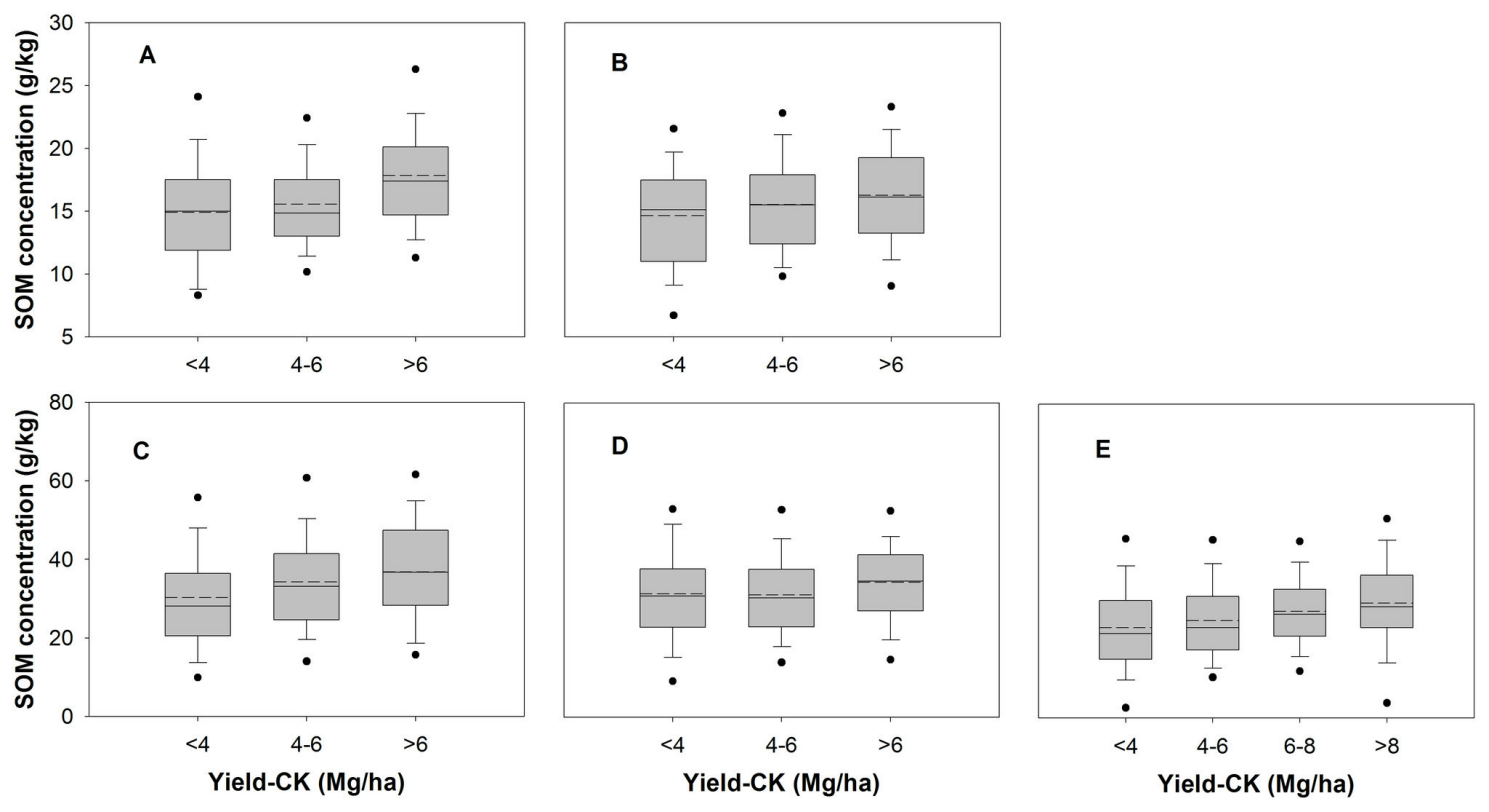
Relations between soil organic matter (SOM) concentrations and grain yield without fertilizer addition for on-farm trials (Yield-CK) in 5 major irrigated cereal-based cropping systems in China. (a) winter wheat in north China (n=354); (b) summer maize in north China (n=425) (c) early rice in south of China (n=697); (d) late rice in south of China (n=688); (e) single rice in Yangtze River Basin (n=2474). Solid and dashed lines in this figure indicate median and mean yield, respectively. The box boundaries indicate upper and lower quartiles, the whisker caps indicate 90th and 10th percentiles, and the circles indicate the 95th and 5th percentiles.

Increase in Yield-CK between 1980s and 2000s can be attributed to many factors, such as improvement of inherent soil productivity, adoption of improved varieties and crop management practices, and nutrient addition from deposition etc. The assumption “trends in Yield-CK on-farm between 1980s and 2000s mainly reflect changes in inherent soil productivity” was tested by computing relationships between yield (Yield-CK and Yield-BMPs) and SOM concentration. The SOM concentration, a critical parameter related to soil productivity [22,23], has indicated a significant increase in top soils under wheat and maize-based cropping systems in north China and rice-based cropping systems in south China since 1980 (Table S1 in File S1) [24-26]. These relationships were computed for five cereal-based cropping systems using 4638 paired data in on-farm trials. Higher Yield-CK was generally obtained in soils with higher average SOM concentration (Figure 1). For example, Yield-CK of < 4 Mg/ha were obtained with the average SOM concentration of 14.1 g/kg for winter wheat, 14.6 g/kg for summer maize, 30.3 g/kg for early rice, 31.4 g/kg for late rice, and 23.1 g/kg for single rice. The average SOM concentration was 20% higher for winter wheat (16.9 g/kg), 11% for summer maize (16.1 g/kg), 22% for early rice (36.9 g/kg), 10% for late rice (34.5 g/kg), and 19% for single rice (27.5 g/kg) for plots with yields > 6 Mg/ha without fertilizer. Furthermore, there were no clear trends between Yield-BMPs and SOM concentration (Figure S2). Therefore, Yield-CK primarily reflects inherent soil productivity, while Yield-BMP is the results of interaction among soil, fertilizer application and varieties.

**Figure 2:**
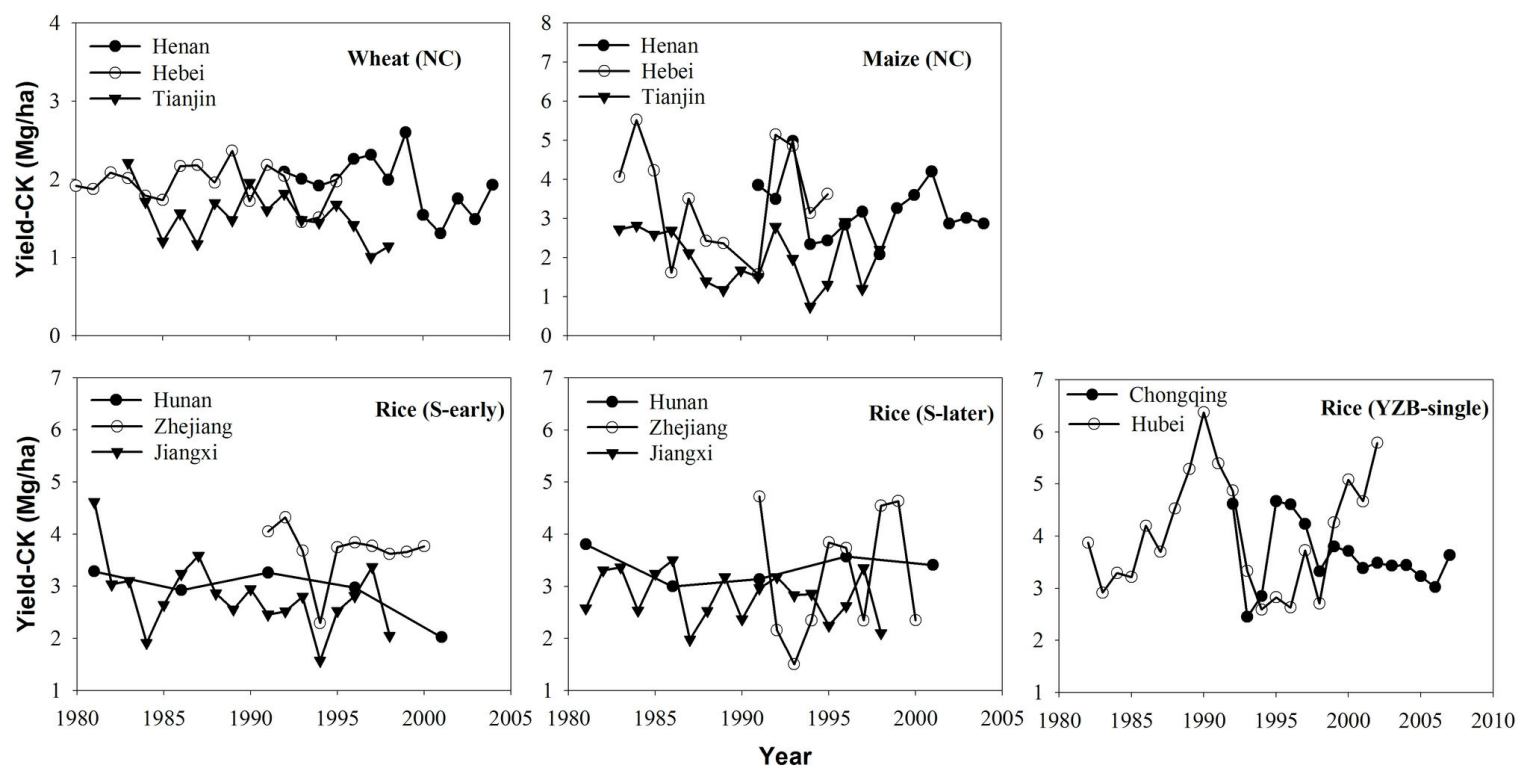
Temporal changes in crop yields in plots without fertilizer addition (Yield-CK) in China’s typical long term experiments of major irrigated cereal-based cropping systems. Note: N, north; S, South of China; and YRB, Yangtze River Basin. See supporting information (Table S1 in File S1) on slopes of each of yield dynamics.

The yield-CK and SOM concentration data for typical major LTEs in irrigated cereal-based copping systems established in the 1980s or 1990s in China were further analyzed. The Yield-CK exhibit stable trends (increase for 2 and decline for others LTEs but with non-significant) in all control plots in these LTEs (Figure 2; Table S2 in File S1). However, SOM concentration showed slight or negligible changes (increase for 3 and decrease for other LTEs with non-significant) (Figure S3), which was not a factor affecting the trend in Yield-CK for these LTEs. While organic and inorganic amendments were not used in control plots for 13-27 years, crop varieties were regularly replaced with the high-yielding and pest-resistant genotypes, and other BMPs were also adopted as and when available. The trends of high Yield-CK maintained over time in these LTEs suggest small to negligible effects of varietals’ improvement since 1980s over two-three decades. Further, subsequent studies show enhanced N deposition over China since 1980s (27), but maintaining yield trends also indicate that increase in N deposition did not measurably increase crop yield even in these LTEs’ control plots.

Therefore, it is highly probable that the increasing trends in Yield-CK under on-farm conditions between the 1980s and 2000s mainly reflect improvements in inherent soil productivity of the predominant irrigated cereal-based cropping systems.

### Effects of improvement in inherent soil productivity on yield and yield stability under BMPs

**Figure 3:**
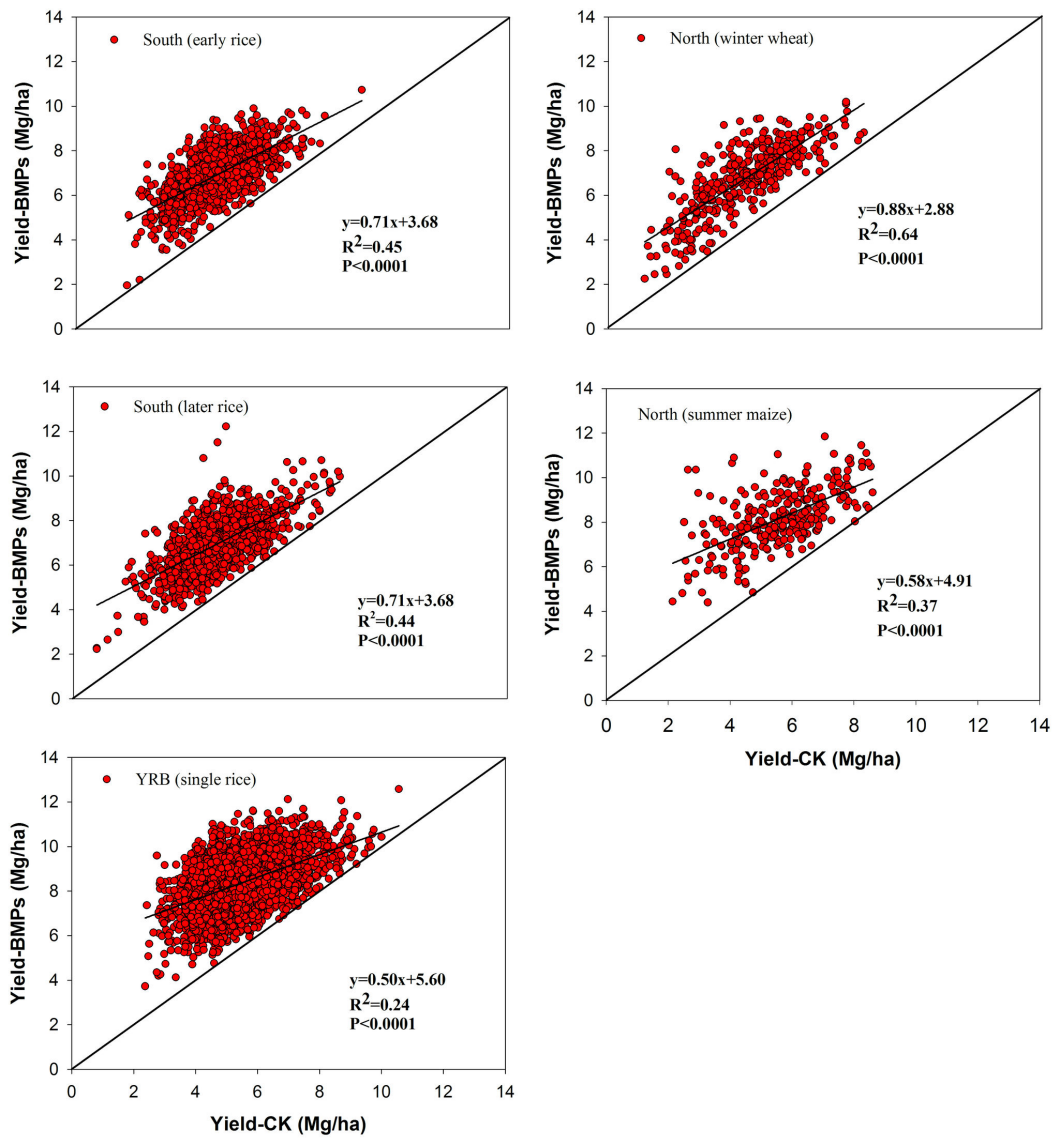
Relations between grain yields without fertilizer addition (Yield-CK) and yields under the best management practices (Yield-BMPs) from on-farm trials in major irrigated cereal-based cropping systems in China. Note: Paired sites involved 621 were for wheat, 457 for maize, 1614 for early rice, 1592 for late rice and 3126 for single rice.

The benefits of improving inherent soil productivity to crop production were assessed by comparing the Yield-CK with Yield-BMPs and its stability in paired plots. The Yield-CK for all five crops were positively and significantly correlated with Yield-BMPs (p<0.0001, Figure 3). Thus, irrespective of fertilizer application, the inherently better soils led to higher cereal crop yields compared with those on poor soils. This trend supports the proverbial Chinese saying that “all boats rise with the rising water”.

**Figure 4:**
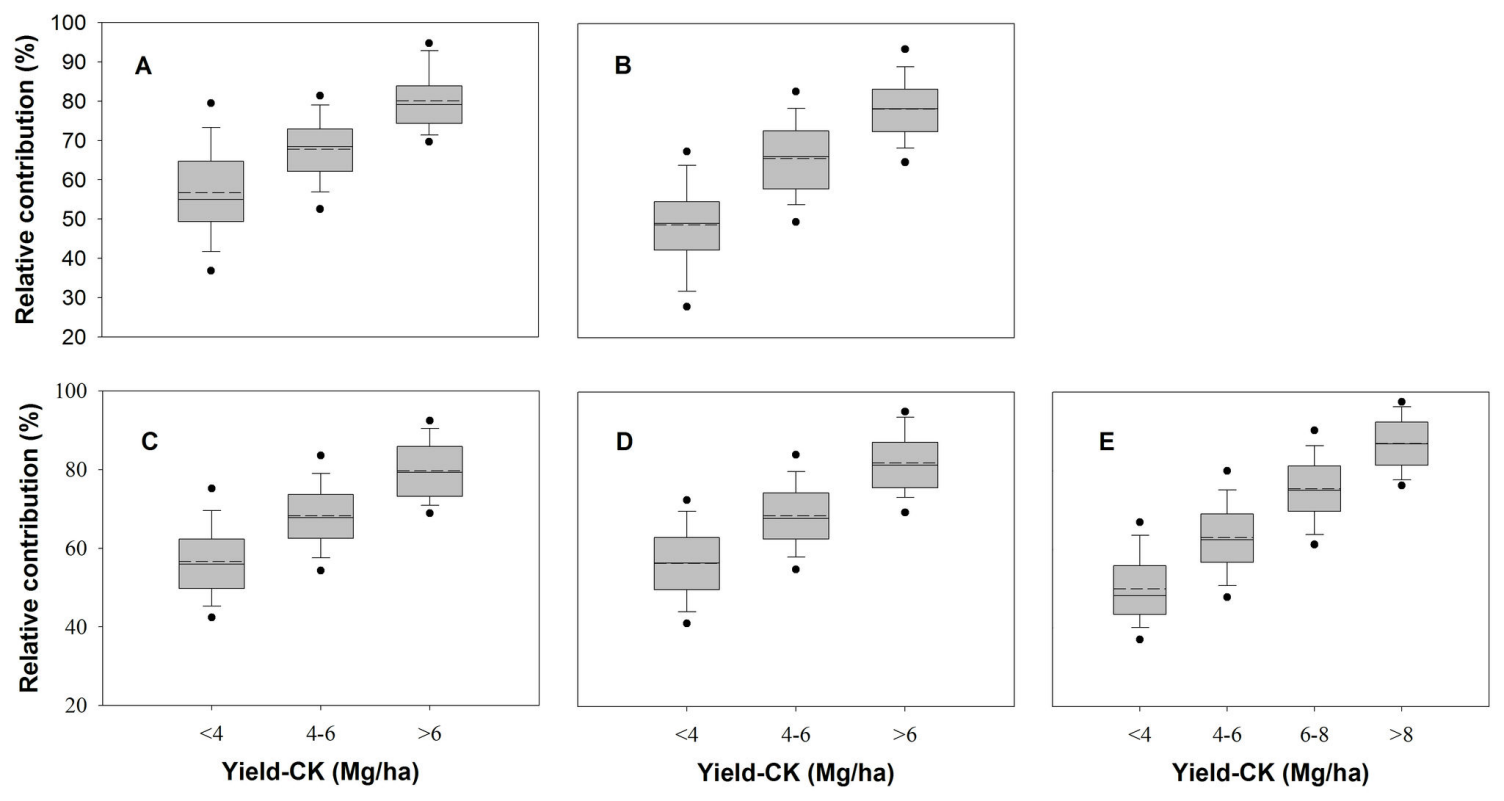
Relative contributions of improvements in inherent soil productivity to yield under BMPs in 5 major irrigated cereal-based cropping systems in China. (a) winter wheat in north China (n=457); (b) summer maize in north China (n=621); (c) early rice in South of China (n=1614); (d) late rice in South of China (n=1592); (e) single rice in Yangtze River Basin (n=3126). Solid and dashed lines in this figure indicate median and mean yield, respectively. The box boundaries indicate upper and lower quartiles, the whisker caps indicate 90th and 10th percentiles, and the circles indicate the 95th and 5th percentiles.

When Yield-CK for all five crops were grouped in increment of 2 Mg/ha over 1980s and 2000s for comparison, the higher yields in control plots led to the higher relative contribution to the total production (the percentage of as yield-BMPs, Figure 4). Increase in Yield-CK from < 4 Mg/ha to > 8 Mg/ha, increased the relative contribution on average from 54% to 79% over five cropping systems. The improvement in inherent soil productivity also increased the yield stability. For example, low Yield-CK was accompanied with higher variability even with adoption of BMPs, especially for those of wheat and maize. However, the variability of yield decreased for all five cropping systems with increase in Yield-CK (Table 3).

**Table 3:** The effects of improvements in inherent soil productivity on yield stability upon adoption of best management practices (BMPs) for China’s major irrigated cereal-based cropping systems.

Region	Cropping system	Yield-CK* (Mg/ha)	Mean yield under BMPs^†^ (Mg/ha)	Variability^‡^ (%)
North	Winter wheat	<4	5.40 (±1.37)	25
		4-6	7.37 (±0.87)	12
		> 6	8.40 (±0.74)	9
	Summer maize	<4	6.92 (±1.49)	22
		4-6	7.86 (±1.16)	15
		> 6	8.94 (±1.05)	12
South	Early rice	<4	6.01 (± 0.95)	16
		4-6	7.15 (± 0.86)	12
		>6	8.30 (± 0.75)	9
	Late rice	<4	6.02 (±1.04)	17
		4-6	7.12 (±0.91)	13
		>6	8.22 (±0.98)	12
Yangtze River Basin	Single rice	<4	7.20 (± 1.18)	16
		4-6	8.21 (±1.10)	13
		6-8	8.92 (±0.94)	11
		>8	10.01 (±0.77)	8

Note: * Yield-CK: grain yields in control plots without fertilizer addition under on-farm trials; ^†^ Data of grain yield under BMPs were showed as average (±std deviation); ^‡^ Variability is calculated according to equation (2).

### Contributions of improvement in inherent soil productivity to cereal crop yields increase

Increase in crop yield since 1980, defined as the difference between Yield-CK in the 1980s and Yield-BMPs in 2000s, ranged from 3.3 to 4.2 Mg/ha (Table 4), which were the results of improvements in inherent soil productivity, advances in crop management technologies (e.g fertilization technology) and crop varietal improvement between the 1980s and the 2000s. Among these factors, improvements in inherent soil productivity (refer to difference between Yield-CK in the 1980s and 2000s) accounted for 43% increase in total production for wheat, 22% for maize, and 31%, 35% and 22% respectively for early and later rice in south China and single rice in the Yangtze River Basin since 1980 (Table 4). Advances in management technology (such as fertilization etc) and improvement in crop variety together contributed to on average of 69% increase in yield since 1980 over those of the irrigated cereal-based cropping systems (Table 4). With the current data, it is difficult to divide the contributions to increase in crop yields since 1980 between advance in management technology and improvement in crop variety.

**Table 4:** Increase in crop yield since 1980 and contributions of improvement in inherent soil productivity for China’s major irrigated cereal-based cropping systems.

			Contributions of IISP and V&TC to yield increase^†^ (%)	
Region	Cropping system	Yield increase* (Mg/ha)	IISP^‡^	V&TC^‡^
North	Winter wheat	4.15	42.5	57.5
	Summer maize	3.34	21.8	78.2
South	Early rice	3.47	31.2	68.8
	Latter rice	3.59	35.3	64.7
Yangtze River Basin	Single rice	3.63	21.8	78.2

Note: * Increase in crop yield since 1980 defined as the difference between Yield-CK in the 1980s and Yield-BMPs in 2000s were calculated by equation (3). ^†^ IISP, Improvement in soil inherent productivity; V & TC, varieties and technology etc’s changes. ^‡^ Contributions of improvements in inherent soil productivity (IISP) to yield increase since 1980 were calculated by equation (4); contributions of changes in varieties and technology (V & TC) to yield increase since 1980 were assessed as the difference between 1 and that the contribution of inherent soil productivity improvement.

## Discussion

### Plant-based assessment changes in inherent soil productivity since 1980

In the current study, inherent soil productivity is defined as an inherent capacity of the soil to perform productive functions, refers to the actual yield of usable vegetation without nutrient-input addition, and reflects the net results of interaction between soil chemical, physical and biological prosperities.

Nevertheless, the assessment of plant-based changes in inherent soil productivity since 1980 may have been confounded by other factors such as varietal improvement, climate change, O_3_ pollution and N deposition etc. However, the trends in Yield-CK in China’s major LTEs (Figure 2; Table S2 in File S1) indicated small if any effect of improved varieties over this period. Similar trends have been observed in other regions of the world. For example, the average yields of grain crops in Sub-Saharan Africa have stagnated at ~1 Mg/ha since the 1960s despite the adoption of modern varieties because the required essential plant nutrients needed by the improved varieties were not supplied [28]. Yet, grain yields (e.g., maize in Malawi) could be doubled or even tripled through combined use of improved varieties and fertilizers [29]. Since introduction of the new cultivars in 1968, several short-straw varieties of wheat grown in the Broadbalk experiment at Rothamsted, UK have also shown no increase in grain yields in fertilizer omission plots. The latter yield 1.5 Mg/ha compared with 6-7 Mg/ha in plots receiving fertilizers (e.g. N,P,K) [30].

The small affects of improved varieties on changes in Yield-CK since 1980s may be attributed to two reasons. First, traditional plant breeding has been done almost entirely for intensive management, such as fumigated soils with high use of fertilizers [31]. This approach has led to selection against traits which allow plants to maintain high net primary productivity (NPP) and agronomic yields under nutrient-deficient conditions (e.g., Yield-CK) [32]. Second, potential yield, (e.g. yield of a cultivar when grown in environments to which it is adapted, without limitation of nutrients and water and with effective control of pests, diseases, weeds, lodging, and other stresses) [33], have stagnated in many regions of the world for major food crops during the past 30 years [4,14]. Similarly, the potential yield of maize in north China has stagnated since the 1980s [34]. The yield potential of rice also has stagnated since the dramatic increases in yields of rice by IR8 released in 1960s, and hybrid varieties introduced in 1970s and 1980s [35,36]. Significant gains in wheat yield in northern China, the winter wheat region, occurred mainly during the early 1980s. When yields of wheat varieties released since 1980 were compared, genetic gain of yield was about 0.5%/yr even with BMPs [37]. Therefore, the stagnating potential yield for the major food crops further implies that the effects of varieties on Yield-CK between 1980s and 2000s may be little, if any.

It is also widely recognized that rapid industrialization since 1980s has increased nutrient (e.g. N) input into agricultural ecosystems, such as through atmospheric deposition [27]. The N deposition has reportedly accounted for 7.5% of the higher NPP from 1980 to 2005 in China [7]. However, the trends of stable Yield-CK in China’s major LTEs do not support the hypothesis that increase in N deposition has contributed to any measurable increase in crop yield. Indeed, N inputs from deposition do not represent a significant net gain to China’s irrigated cereal-based cropping systems. However, other factors such as climate change and air pollution may offset any positive effects of increase in N deposition on crop yield. For example, on national basis, a 4.5% reduction in wheat yields has been attributed to an increase in ambient temperatures between 1979 and 2000 [38]. Similar declining trends have been reported in yields of maize between 1979 and 2002 [39]. Increases in average night-time temperature also have a negative effect on rice yield in the tropics [40]. Tropospheric O_3_ pollution decreased the NPP of crops by about 10.7% [7]. The currently observed regional haze may reduce yields of wheat and rice by 5 to 30% in China’s highly productive eastern agricultural regions [5]. Further, any adverse effects of climate change and O_3_ pollution on crop yield may have been underestimated, because of improvements in inherent soil productivity and resilience.

### The causes of inherent soil productivity improvement on farm in major irrigated cereal-based cropping systems

The observed improvement in inherent on-farm soil productivity of China’s major irrigated cereal-based cropping systems may partly be due to increase in SOM concentration. During the three decades since 1980, SOM concentration in surface layer of soils in most cereal-based cropping systems followed an increasing trend (Table S1 in File S1) [7,24-26]. A dependence of Yield-CK on SOM concentration on farm (Figure 1) indicate that increase in SOM causes a measurable improvement in inherent soil productivity in major irrigated cereal-based cropping systems since 1980. This trend may be attributed to the positive effects of SOM concentration on soil aggregation and tilth, root system development, water-holding capacity, water inﬁltration rate, and the availability of essential plant nutrients such as N, P, S Zn etc [41,42].

At national scale, several factors might have contributed to the observed increase in SOM concentration. First, increase of SOM concentration in most arable lands in China between the 1980s and 2000s is attributed to the adoption of the “Green Revolution” technologies, which progressively enhanced crop production and increased the input of root exudates and root biomass, and thus the SOM concentration [24,26]. For example, between 1980 and 2007, average yields of rice, maize, and wheat increased by 53%, 76%, and 153%, respectively; (values were calculated from data in China Agriculture Yearbook). Second, retention of crop residues on farmland (especially since the 1990s when modern household fuel replaced traditional biogenic resources) and application of organic manure also enhanced the SOM concentration in soils of major irrigated cereal–based cropping systems [24,26].

The SOM concentration, a critical parameter related to soil productivity [22,23], has been considered as the surrogate to test the first hypothesis in the current study. The approach of plant-based estimation of inherent soil productivity is reflected in the net results of interaction between soil chemical, physical and biological prosperities. Nonetheless, enhancement of other soil-related factors may also be responsible for the observed improvement in inherent soil productivity. For example, low soil available P (Olsen-P <10 mg/kg) had been an important factor limiting crop production in the calcareous soils in the north and the paddy soils in the south of China in 1980s [43]. However, because of the long period of P fertilization on arable land, more than 37 million metric tons of (Mt) P accumulated in soil, resulting in an increase in average soil Olsen P from 7.4 to 24.7 mg/kg between 1980 and 2007 [44]. Further, it should be noted that the measured improvement in inherent soil productivity by plant based approach in the current study do not exclude the certain form of soil degradation for those cropping systems such as soil acidification [18] and reduction in thickness of topsoil [1,19]. Additional research is therefore required for a thorough understanding of the specific or multiple soil properties in determining differences in inherent soil productivity of these major irrigated cereal-based cropping systems.

In addition, China implemented several programs during 1970s and 1980s for land improvements in the Huang Huai Hai Plain (North China) and in the South Red Hills region, and for water and soil conservation in the Loess Plateau in North and Northwest China since the 1950s. Technological interventions included land leveling, and establishment of terraces and water conservation facilities [45,46]. These restorative programs played an important role in alleviating principal soil-related constraints on arable land.

Thus, adopting a comprehensive strategy of improving inherent productivity of soils in China, through combination of engineering (e.g. land leveling, and establishment of terraces and water conservation facilities) and biological (e.g. increase input) approaches, may be an important lesson for the other developing world (e.g. South Asia, Sub-Saharan Africa, central America, the Andean region, and the Caribbean).

### Further improvements in inherent soil productivity for advancing China’s food and environmental security

Following the “Green Revolution,” agricultural intensification brought about a quantum jump in crop yields in China [1,4]. However, the analysis presented herein clearly indicates the importance of improving the quality of soils to increase crop productivity. Indeed, the so called “Agricultural Miracle in China” would not have happened without improvement in inherent soil productivity.

Future food demand for China must be met through intensification of agronomic production on an ever-dwindling cropland area and without compromising the environmental integrity. Such intensification of crop production will depend on good quality soil [1]. Soils of inherent good quality produce high and stable yields because of resilience to biotic and abiotic stresses (Table 3, Figure 3 and Figure 4). Therefore, China must adopt a strategy of continuously improving soil quality and agronomic productivity. This strategy is also necessitated because of a limited potential of additional improvements through engineering-based measures such as land leveling, terracing, and restoring salinized soil, etc. Improving soil productivity through better management of SOM may provide multiple benefits [47], because as much as two-thirds of Chin’s soils are still impoverished and have lower SOM concentration then ecological potential.

### Concluding remarks

The current study assessed temporal changes in inherent soil productivity and its contributions to drastic increase in China’s cereal crop yield by using a plant-based agronomy approach. The data show that increase in Yield–CK under on-farm conditions between 1980s and 2000s reflect primarily improvements in inherent soil productivity. The latter contributed on average to 31% yield increase for China’s major irrigated cereal-based cropping systems since 1980. However, additional research is needed for a thorough understanding the importance of either a specific or multiple soil prosperities to inherent soil productivity, and to quantify the effects of different variables underpinning crop and regional differences in Yield-BMPs. Such information would be useful to establish guidelines for bringing about further improvements in agronomic yields through management of soil, genotypes, inputs, and the environment.

The experiences in China have a wider relevance at global scale. As the world’s most populous country, China impacts global food supply and demand, and the use of natural resources. Food insecurity affects 850 million people worldwide, which remains a major challenge globally [48,49]. The solutions for attaining global food security are scientific and technological (e.g., high-tech seeds and better water and nutrient management) [10,48], political (e.g., maintaining good governance) [50], economic (e.g., investment in agriculture research and education, rural infrastructure) [51], and ethical (e.g., changing diet preferences, gender and social issues) [52]. However, increasing agronomic yields on existing cropland with less resource use (e.g., nutrient and water) and environmental protection are among major goals [48,49]. This study highlights that improvement in inherent soil productivity are likely to give a large and long-term return towards advancing food production for the growing human population and rapidly changing climate.

## Supporting Information

File S1 **Tables S1 & S2, and references.**
(DOC)

Figure S1 **Geographical distribution of collected data.**
(TIF)

Figure S2 **Relations between soil organic matter (SOM) concentrations and grain yield under best management practices on-farm trials (Yield-BMPs) in 5 major irrigated cereal-based cropping systems in China, references S1 to S151 in File S1.** (a) winter wheat in north China (n=354); (b) summer maize in north China (n=425); (c) early rice in south of China (n=697); (d) late rice in south of China (n=688); (e) single rice in Yangtze River Basin (n=2474). Solid and dashed lines in this figure indicate median and mean of yield, respectively. The box boundaries indicate upper and lower quartiles, the whisker caps indicate 90th and 10th percentiles, and the circles indicate the 95th and 5th percentiles.
(TIF)

Figure S3 **The changes in SOM concentration in control plots in long term experiments in major irrigated cereal based cropping systems, references S221 to S226 in File S1.**
(TIF)
