# Supplementary material for: Plant-Based Assessment of Inherent Soil Productivity and Contributions to China’s Cereal Crop Yield Increase since 1980
Source: PLoS One. 2013 Sep 18;8(9):e74617. doi: 10.1371/journal.pone.0074617 (PMC3776784; doi:10.1371/journal.pone.0074617)
Supplement: File S1 — Tables S1 & S2, and references. (DOC) [file pone.0074617.s001.doc]

Supporting online materials

Supporting Figure Legends

Figure. S1 Geographical distribution of collected data.

Figure S2. Relations between soil organic matter (SOM) concentrations and grain yield under best management practices on-farm trials (Yield-BMPs) in 5 major irrigated cereal-based cropping systems in China, reference from S1 to S151. (a) winter wheat in north China (n=354); (b) summer maize in north China (n=425); (c) early rice in south of China (n=697); (d) late rice in south of China (n=688); (e) single rice in Yangtze Rive Basin (n=2474). Solid and dashed lines in this figure indicate median and mean of yield, respectively. The box boundaries indicate upper and lower quartiles, the whisker caps indicate 90th and 10th percentiles, and the circles indicate the 95th and 5th percentiles.

Figure S3. The changes in SOM concentration in control plots in long term experiments in major irrigated cereal based cropping systems, reference from S221 to S226.

Table S1. Changes in soil organic matter (SOM) concentrations of crop lands in China’s major irrigated cereal-based cropping systems since 1980^†^.

| Region | Cropping system | 1980s | 2000s | Increase |
| --- | --- | --- | --- | --- |
| North | Winter wheat-Summer maize | 9.5（±2.8） | 14.2（±2.6） | 4.7（±2.2）** |
| South | Double rice | 29.2 (±6.0) | 32.5 (±4.9) | 3.3 (±4.7) * |
| Yangtze River Basin | Single rice | 20.2 (±8.0) | 23.4 (±8.7) | 3.2 (±4.0) ** |

Note: p=0.01-0.05; **, p<0.001; data were showed as average (±std deviation);

† To objectively evaluate the changes in SOM between 1980s and 2000s in different cropping systems, 68 articles (reference S152-S220) were selected on the basis of the following criteria: (1) the study period is from the early 1980s to 2000s, if articles for this period are not available for some regions, other articles which reported changes in SOC/SOM from 1980s to 1990s were selected; (2) the data on SOC/SOM concentration for 1980s and 2000s (or 1990s) are available; (3) the baseline is represented by the measurements at the provincial-scale. If articles on a provincial-scale are not available, the data from other published articles following the same criteria in these provinces were summarized to estimate changes in SOC/SOM concentration between 1980s and 2000s; (4) only changes in SOC/SOM in topsoil (0-20cm depth) were considered. More than 140,168 soil samples and/or sampling sites were included in these selected articles. The conversion factor of 1.742 was used to convert SOC into SOM concentration.

Table S2. Summarized results of a linear model analysis for wheat, rice and maize yield dynamics (Mg/ha) in control plots in major LTEs since experiment established, reference from S221 to S226.

| Region | Cropping systems | Sites | Slope | R^2^ | P* |
| --- | --- | --- | --- | --- | --- |
| North | Winter Wheat | He nan | -0.041 | 0.20 | 0.12 |
|  |  | He bei | -0.011 | 0.045 | 0.43 |
|  |  | Tian jin | -0.030 | 0.20 | 0.079 |
|  | Summer Maize | He nan | -0.043 | 0.053 | 0.43 |
|  |  | He bei | 0.023 | 0.005 | 0.82 |
|  |  | Tian jin | -0.058 | 0.15 | 0.14 |
| South | Early Rice | Zhe jiang | -0.018 | 0.01 | 0.78 |
|  |  | Hunan | -0.050 | 0.58 | 0.13 |
|  |  | Jiang xi | -0.056 | 0.20 | 0.066 |
|  | Later Rice | Zhe jiang | 0.67 | 0.28 | 0.64 |
|  |  | Hunan | -0.0044 | 0.011 | 0.86 |
|  |  | Jiang xi | -0.027 | 0.071 | 0.32 |
| Yangtze River Basin | Single Rice | Chong qin | -0.0384 | 0.08 | 0.28 |
|  |  | Hu Bei | 0.030 | 0.028 | 0.47 |

* Statistic significance at P < 0.05 level

**References**

S1. W.F. Qin, Effect of nitrogen and phosphorus fertilizer application on grain yield and quality of winter wheat. *J. Agric.Hebei* **11(4),** 10-18 (1988).

S2. A.L. Shen, S.F. Zhang, G.Y. Gong, G. Li, Study on optimal fertilization and nutrient balanced by wheat in rainfed lands. *Agric. Res. Arid. Areas* **11(4),** 36-40 (1993).

S3. J.Q. He, A study on economical supply of phosphorus-nitrogen fertilizer in the sand concretion black soil. *J. Huazhong Agric. Univ*. **6,** 58-65 (1989).

S4. D.M. Huang, H.J. Ji, Q.M. Xu, S.M. Wu, S.H. Liu, Using yield response model in determining nitrogen and phosphorus application for wheat. *Beijing Agricul. Sci.* **4(5),** 231-234 (1982).

S5. D.M. Huang, Z.C. Wang, H.Q. Ding, Q.L. Sun, Research on the optimum ratio of nitrogen and phosphorus fertilizer application for wheat at Beijing suburbs. *Soil Bull.* **4,** 66-70 (1983).

S6. Z.S. Zhao, Effects of nitrogen and phosphorus fertilizer integrated application on wheat yield. *Soil Fert.* **2,** 29-30 (1983).

S7. M.G. Zhuo, A discussion on appropriate ratio of nitrogen, phosphorus and potassium fertilizer application for crops. Acta .*Baiquan. Agric. Colg.* **11(1),** 80 (1983).

S8. F.M. Chi, D.B. Wei, Z.L. Wang, Relationship between the different ratios of nitrogen, phosphorus and potassium application and the formation of wheat yield. *Shandong Agric. Sci.* **1,** 6-10 (1984).

S9. G.L. Zhang, D.J. Bao, S.Z. Dou, Study on effect of nitrogen, phosphorus and potassium ratio on the wheat yield in middle and low fertility soils. *Henan Agric. For. Sci. Technol*. **8,** 4-7 (1984).

S10.D.F. Zuo, Research on efficiency of several major nitrogen fertilizers types such as ammonium bicarbonate fertilizer through fertilizer response model. *Fert. Ind.* **2,** 23-26 (1984).

S11.S.M. Zhang, Y.Z. Liu, Z.Q. Chen, Effect of increase nitrogen, phosphorus and potassium fertilizer application on nutrition components of wheat grain. *Shandong Agric. Sci.* **1,** 11-13 (1984).

S12.S.L. Lin, H. Yan, S.H. Zhao, Preliminary report on indexs of nutrient abundance-deficiency in Shandong brown soil. *Shanxi Agric. Sci.* **1,** 15-18 (1985).

S14.Z.Y. Liu, Z.M. Li, X.Y. Wang, Preliminary report on nitrogen and phosphorus rates experiment in wheat in hilly upland in west of Henan. *Henan Agric. Sci*. **9,** 4-5 (1986).

S15.S.C. Yang, Z.R. Sun, X.Q. Liu, The optimum nitrogen and phosphorus fertilizer rates for wheat in different soils at Huanghuaihai Plain. *Soil Fert.* **1,** 1-5 (1988).

S16.L.P. Guo, X.R. Wang, F.S. Zhang, X.P. Chen, D.R. Mao, Effect of fertilizer application in different years on crop yields and fertilizer recovery. *J. Chin. Agrometeorol* **20(4)**, 20-23 (1999).

S17.S.D. Ji, T.F. Chen, H.F. Shi, Effects of nitrogen and phosphorus fertilizer application ratio on grain yield and quality of winter wheat. *Acta. Henan Vocational technol. Normal Colg*. **17(3-4)**, 94-98 (1989).

S18.X.H. Yang, W.H. Tang, H.Q. Lei, X.J. Li, Spectroscopic diagnostics and estimations of nitrogen abundance and deficiency of wheat under nitrogen and phosphorus interaction. *Remot. Sen. Inf.* **1,** 30-34 (1992).

S19.W.L. Rui, S.M. Zhang, C.C. Guo, Fixed position experiments of fertilization on loamy alluvial soil in south of Shandong. *Soil Fert.* **5,** 30-32 (1996).

S20.Q.Y. Li, J.H. Shi, Basic experiences of wheat production in upland at Wanrong county. *Shanxi Agric. Sci.* **8,** 11-17 (1981).

S21.G.B. Yang, P. Da, F. Liu, Effects of different rate and ratio of nitrogen,phosphorus and potassium on winter wheat yield. *Shanxi Agri. Sci.* **4,** 12-16 (1982).

S22. Y.K. Gao, Research on effects of nitrogen and phosphorus fertilizer application in three-level lands. *Shanxi Agric. Sci. Technol.* **6,** 11-13(1990).

S23. K,G, Sun, G.H. Jia, X.F. Meng, Effects of application of silicon-potassium fertilizer on yield of winter wheat and cotton. *Agric. Sci. Technol.* **(6),** 12-15 (2003).

S24. X.R. HuangPu, et al.,Effects of long-term located fertilization on yield and quality of High-gluten wheat Zhengmai 9023. *J. Henan Agric. Sci.* **(4),** 77-80 (2006).

S25.S.Q. Zhai, C.L Li, Y. He, X.G. Liu, X.Y. Wang, Study on the effects of N and P fertilizer on yield of the high quality wheat. *J. Henan Vocation. Tech. Teach. Colg.* **32(3),** 6-8 (2004).

S26.T.R. Yang, S.B. Zhang, J. Fang, C.J. Liu, Q. Zhao, Effect of formulated fertilizer application on wheat yield. *J. Hebei Agric. Sci*. **11(1),** 66-67 (2007).

S27.Annual report of major demonstration counties of soil test and fertilizer recommendation project for wheat. 2006-2009.

S28. S. C. Yang, Z.R. Sun, X.Q. Liu, Studies on the optimum nitrogen and phosphorus fertilizer rates for corn and nitrogen balance in soil-crop system in different soils of Huanghuaihai Plain. *Soil Fert*. **3,** 6-13 (1988).

S29. R.D. Wu, The study on effects of integrated nitrogen and phosphorus fertilizer application on crop performance in dry calcareous soils. *Soil Fert.* **3,** 27-29 (1981).

S30. X.R. Li, Effects of nitrogen and phosphorus fertilizer integrated application on maize yield and economic efficiency. *Tianjin Agric. for. Sci. Technol.* **4,** 11-13 (1991).

S31. J.Q. He, A study on economical supply of phosphorus-nitrogen fertilizer in the sand concretion black soil. *J. Huazhong Agric. Univ*. **6**, 58-65 (1989).

S32. S.M. Zhang, Y.Z. Liu, Z.Q. Chen, R.Z. Cui, Effects of interaction between nitrogen and phosphorus, nitrogen and potassium fertilizer on summer corn. *Soil Fert*. **2,** 27-30 (1989).

S33. Y.S. Fan, Y.J. Wang, D.H. Su, A study on fertilization for high yield summer corn. *Soil Fert.* **3,** 32-35 (1990).

S34. W.L. Rui, S.M. Zhang, C.C. Guo, Fixed position experiments of fertilization on loamy alluvial soil in south of Shandong. *Soil Fert.* **5,** 30-32 (1996).

S35. B.S. Li, Y.X. Wang, Research on the index and parameters of economic fertilization for summer corn. *Tillage Cult*. **4,** 51-53 (1993).

S36. Q.H. Chu, X. Xu, T.H. Shao, Different soils’ physical and chemical characteristics and their responses to fertilizer application in Shanxi. *Agric. Res. Arid. Areas* **3,** 45-51 (1988).

S37. Y.C. Wei, J.P. Yan, Effects of nitrogen nutrition on growth and development of major organs of corn. *Acta. Baiquan Agric. Colg.* **11(2),** 1-6 (1983).

S38. J.Q. Zhang, W.M. Wang, G. Cao, S.P. Zhang, N.K. Feng, Study on fertilization and water use efficiency of corn in semi-humid areas. *Tunliu County Soil Fert.* **6,** 11-13. (1990).

S39. Annual report of major demonstration counties of soil test and fertilizer recommendation project for maize. 2006-2009.

S40. H.R. Yu, The study of N rate for summer maize on various soil fertility for Fluvo-aquic in Zhoukou , *Mod. Agric. Sci. Techno.***11**: 47,50

S41. S.M.Zhang, H.C. Zhang, The study on fertilization parameter for summer maize in Brunisolic soil region. *Hebei Agric. Sci. Techno.* **21:** 12-15 (2008)

S42. H.J. Huai, H.L. Zhang，W.T. Cai，F. Chen. Effect of different nitrogen rates on nitrogen utilization and residual soil nitrate of spring maize. *J. Agro—Envren. Sci.* **28(12):** 2651-2656 (2009)

S43. F. Gao, B.Y. Yang, S.C. Wang, G.L. Wang, Z.P. Peng, S.C. Xue, S.H. Wang. Preliminary study on the optimal application of N,P and K in maize under different soil fertility. *Journal of Agricultural University of Hebei,* **31(3):**17-20, 36 (2008)

S44. J.L. Gao, et al. Preliminary study on decreased application of nitrogen and phosphorus fertilizers on maize in fertile soil in Pingdu city. *Shandong Agric. Sci.* **2**, 67-69 (2010)

S45. Q.S. Zhu, The study on nutrient supply capacity and response to fertilization in Yellow paddy soil. *Hubei Agric. Sci.* **10,** 16-18 (1981).

S46. Effect of various rates and ration of nitrogen, phosphorus and potassium fertilizer application on rice yield---a summary of fertilizer experiment net of Jiangxi province. *J. Jiangxi Agric. Sci. Technol*. **12,** 9-12 (1987).

S47. Z.Y. Zhang, J. Yang, Study on integrated nitrogen, phosphorus and potassium fertilizer application for rice. *J. Guangxi Agric. Sci.* **7**, 19-21 (1982).

S48. L.C. Hong, A summary of fixed position rice field experiment with different rates and ration of nitrogen, phosphorus and potassium fertilizer application. *J. Jiangxi Agric. Sci. Technol.* **11,** 12-14 (1985).

S49. Research on integrated nitrogen, phosphorus and potassium fertilizer application for rice. *J. Guangxi Agric. Sci.* **3,** 37-43 (1984).

S50. X.Y. Liang, The effect of big granular urea on rice. *Fert. Ind.* 3, 28-61 (1992).

S51 Y.J. Lu, Q.H. Liu, The influence of optimum fertilization on rice yield and soil fertility. *J. Hubei Agric. Sci.* **6,** 36-41 (1994).

S52. Z.H. Yang, S.Y. Zhuang, Y.H. Liu, Y. Liang, Effects of organic and inorganic combination application on rice grain yield and quality. *J. Fujian Agric. Sci. Technol.* **1,** 17-18 (1993).

S53. F. Liu, X.W. Zhang, C.S. Wang, Study on the response of long term fertilization in double rice cropping system of southern of An Hui. *Plant Nutr. Fert. Sci*. **4(3)**, 224-230 (1998).

S54. J.G. Peng, Z.D. Zheng, Z.Q. Lin, Study on fertility characteristics, improvement strategy of yellow paddy soil. *Acta. Fujian Prov. Acad. Agric. Sci.* **1(2)**, 8-15 (1986).

S55. W.L. Zhu, Primary study on nutrient abundance-deficience index in paddy soil. *Soils.* **5,** 259-263 (1988).

S56. Q. C. Hu, Y.T. Ye, K.F. Le, Z.Q. Huang, Effects of ratio and amount of nitrogen, phosphorus and potassium fertilizer application on rice. *Soils*. **2,** 87-91 (1994).

S57. X.S. Guo, X.W. Zhang, S.Y. Ye, Study on the contribution of indigenous nutrient supply capacity and optimum fertilization to paddy rice. *J. Anhui Agric. Sci.* **22(1),** 41-44 (1994).

S58.Y.B. Zou et al. Effects of one-time application of different formula fertilizer on the yield and quality of high quality rice. *Rev.Chin. Agric. Sci. Technol.* **5(4)**, 36-41 (2003).

S59. P.A. Dai, J. Nie, S.X. Zheng, J. Xiao, Efficiency of nutrient utilization of controlled-release nitrogen fertilizer for rice at different soil fertility levels. *Chin. J. Soil. Sci.* **34(2),** 115-119 (2003).

S60. Y.B. Zou, Introduction and application of rice seedling quantitative measure fertilization technology. Chin. *Agric. Technol. Extension* **22(7),** 35-37 (2006).

S61. L.Z. Ma, Z. Quan, Z.L.Chen, Test the effects of nitrogen, phosphorus and potassium fertilizer on later rice. *Guangdong Agric. Sci.* **9,** 34-35 (2006).

S62. S.L. Li et al., 2007. Study on residual effect of nitrogen in flow aquic paddy soil. *Hubei Agric. Sci.* **46(5),** 727-729 (2007).

S63. K.M. Fang, G.M. Zhong, M.S. Zhang, Study on effect of different ratio of nitrogen, phosphorus and potassium on yield of rice. *Acta. Agric.Jiangxi* **19(10)**, 50-53 (2007).

S64. G.Q. Zhang, Q. Chen, D.W. Li, S.Q. Zhao, Study on the effect of fertilizer on late japonica Xiushui 110. *Mod. Agric. Sci. Technol.* **14,** 114 (2007).

S65. C.L. Yu, Z.F. Feng, Discussion on nitrogen, phosphorus and potassium rates for rice in water channel plain area. *Zhejiang Agric. Sci*. **4,** 442-443 (2007).

S66. Z.X. Guo, Z.G. Qiu, Study on nitrogen, phosphorus and potassium fertilizer combined application in sandy-muddy field. *Guangxi Agric. Sci.* **39(2),** 206-209 (2008).

S67. Z.Z. Wu, J.H. Mo, Study on "3414" fertilizer field experiments for rice. *Gansu Agric.* **6,** 93-95 (2008).

S68. Y.H. Yang, Y.X. Qiu, D.X. Li, Q.Y. Huang, Fertilization research on rice in middle and lower yield sandy-mud paddy field. *J. Guangxi Agric.* **23(4),** 5-8 (2008).

S69. Y.G. Gao et al., Research on the effects fertilizer on later-cropping hybrid rice. *Soil Fertil Sci. China.* **2,** 48-51 (2008).

S70. W.N. Wang, Y.Y. Wang, Z.Q. Yao, The “3414” fertilization effect and fertilizer rate recommendation in late-season rice. *Hubei Agric. Sci.* **48(2),** 306-309 (2009)

S71. X.S. Ke, Q. Chen, D.W. Li, S.Q. Zhao, Effects of fertilization on rice at new land consolidation area. *Mod. Agric. Sci. technol.* **(6),** 139-143 (2009).

S72. Y.J. Li, Y.X. Qiu, L. He, Z. Liu, Study on “3414” fertilizer response experiment for hybrid rice in middle and lower yield field. *Mod. Agric. Sci. technol*. **2,** 121-124 (2009).

S73. Annual report of major demonstration counties of soil test and fertilizer recommendation project for rice in Jiangxi. 2006-2009.

S74. L.Z. Zeng, H.W. Zeng, Z. Zeng, Preliminary report of experiment on rice formula fertilization with soil test at Xingning city. *Guangdong Agric. Sci.* **(4),** 60-61 (2009).

S75 Y.C. Fan, Q.R. Tao, Integrated fertilization technology for rice. *Jiangxi Sci.* **3(4),** 47-54(1985)

S76. F. Wang, T. Luo, Study on remediation of rice phosphorus deficiency stress by formula fertilizer application. *Fujian Rice Technol.* **20(3)**, 18-19 (2002).

S77. S.Q. Dong, Effects of the different ratio of nitrogen, phosphorus and potassium on rice in the“3414” field experiment. Chin. *Counfryside Well-off Technol.* **12**, 60-62 (2006).

S78. X. Huang et al., Effect of formula fertilization by soil testing on growth and yield of rice. *J. Guangdong Agric. Sci.* **5,** 47-57 (2008).

S79. S.L. Cheng, Eeffect of formula fertilization by soil testing on early rice. *J. Hebei Agric. Sci.* **12(4)**, 55-57 (2008).

S80. M.F. Wu, Y.Z. Wang, W.H. Wang, Y.D. Lai, K.H. Guo, Study on the fertilizer efficiency for rice in Danzhou Hainan Province. *Chin. Agric. Sci. Bull.* **24(7)**, 280-283 (2008).

S81. L.W. Lv, T.Z. Wang, Experiment of "3414" balance fertilization for rice. *Anhui Agric. Sci. Bull.* **14(23)**, 138-139 (2008).

S82. X.R. Ye, Z.L. Deng, C.Y. Zhu, Study of formula fertilization by soil test for rice in the field experiment at Nanxiong city. *Guangdong Agric. Sci.* **4**, 57-59 (2009).

S83. L.Z. Zeng, H.W. Zeng, Z. Zeng, Preliminary report of experiment on rice formula fertilization with soil test at Xingning city. *Guangdong Agric. Sci.* **(4)**, 60-61 (2009).

S84. B. Zhou, Influence of nitrogen management on yield and dry matter accumulation of early rice. *J. Hebei Agric. Sci.* **11(3)**, 14, 20 (2007).

S85. A.P. Wang, J. L. Deng, Studying the impact of biological organic fertilizer on the rice yield and quality. *Crops.* **5**, 28-30 (2006).

S86. Y.Y. Li et al., Response of fertilization to early rice performance with super-high yielding cultivation. *Anhui Agric. Sci. Bull.* **35(23)**, 7215- 7218 (2007).

S87. L.J. Zheng, G.Y. Zeng, P.F. Wang, Influence of organic fertilizer, chemical fertilizer long-term combination application on the rice yield and soil nutrient. Chin. *Agric. Sci. Bull.* **17(3)**, 48-50 (2001).

S88. R.F. Wang, D.M. Huang, Y. Cui, The Report on soil fertility monitoring in different areas of China Ⅱ. The trend of soil fertility changes and the reasons analysis in double rice cropping system of Southern China. *Soil Fert.* **2**, 3-8 (2002).

S89. J.G. Wu, D.R. Luo, Y.W. Ning, Y.Z. Jiang, Evaluation in efficiency of choride-bearing fertilizer applied in farm land. *Acta. Pedologiga. Sinica.* **32(3)**, 321-326 (1995).

S90. J.A.Shao et al. The role of organic and chemical fertilizer combination application in rice yield increase. *J. Shanghai Agric. Sci. Technol.* **3**, 17 (1994).

S91. G.L. Chu, G.Q. Zhan, J.Y. Ding, A study on rate and ratio of nitrogen, phosphorus and potassium fertilizer application for rice in hilly region. *J. Hubei Agric. Sci.* **3,** 5-7 (1996).

S92. Y. Wang, Appropriate rate and ratio of nitrogen, phosphorus and potassium fertilizer for rice --- the second report of field experiments. *Fert. Ind.* **3**, 15-21 (1985).

S93. S.L. Zhang, B. Yin, D.S. Yu, in: Basic research on nitrogen behavior and efficient utilization in major agoecosystems in China (eds Z.L. Zhu, F.S. Zhang) 232 (Science press, Beijing, 2009).

S94. A.Y. Liu, Research on nitrogen precising application technology for rice in Jiasha soil with different fertility. *J. Shanghai Agric. Sci. Technol.* **5**, 46-47 (2008).

S95. J.X. Xie, J.S. Wu, H.F. Tan, J.D. Wang, Y.C. Zhang, Influence of different ratio of nitrogen, phosphorus and potassium on nutrient uptake and yield of rice. *Jiangsu Agric. Sci.* **5**, 258-260 (2008).

S96. D.J. Wang, J.h. Li, R.J. Sun, L.Z.Xia, G. Lian, Optimum nitrogen rate for a high productive Rice-wheat system and its impacts on the groundwater in the Taihu Lake area. *Acta Pedologzca Sinca,* **40(3)**, 426-432(2003).

S97. Z.G. Sun, Influence of different nitrogen, phosphorus and potassium fertilizer ratio on rice yield and benefit. *Agric. Equipment Technol.* **31(4),** 30-31 (2005).

S98. G.L. Zhang, D.P. Chen, M. Luo, Influences of fertilization formula on rice population quality and yield. *J. Anhui Agric. Sci.* **33(12),** 2277- 2278 (2005).

S99. Y.F. Tao, The effect of nitrogen long-term synergistic agent on rice production. *J.Shanghai. Agric. Sci. Technol.* **4**, 97-98 (2003).

S100. Y.Q. Wang et al., Influences of rape straw return and fertilizer management on the growth of rice. *J. Anhui Agric. Sci.* **37(11)**, 4923-4924 (2009).

S101. X.M. Zhang, X.S. Guo, Effect of rational fertilization on growth and development in hybrid Japonica rice. *Reclaiming Rice Cult.* **3**, 48-51 (2007).

S102. S.F.Wang, H.l. Chen, H. liu, The effects of Nitrogrn,Phosphorus and potassium balance application on rice yield and fertilizer use efficiency, *Till. Cult.* **3**, 38-30 (2002).

S103. H.B. Ye, X.P. Huang, M. Yao, Y. Yin, Influence of different formula nitrogen and potassium on rice N use efficiency. *J. Shanghai Agric. Sci. Technol.* **3**, 38-39 (2003).

S104. D.Q. Liu, W.B. Chai, Z.B. Gao, Study on balanced fertilizer application and parameters calculated for rice fertilization. *J. Anhui Agric. Sci.* **31(4)**, 672-673 (2003).

S105. J.J. Zhao, Effect of application quantity of nitrogen, phosphorus and potassium fertilizer on resistant capability of rice against hot disaster of high temperature. *Soil Fert*. **5**, 13-16 (2005).

S106. H. Fei, Y.J. Dong, Study on the optimum fertilizer on Hybrid rice. *Rural Econ. Sci. Technol.* **10**, 83-84 (2006).

S107. H.Gao, H.C.Zhang, Q.G.Dai, D.J.Zhu, C.Y.Hu, The effects of various soil fertilty and fertilizer rates on rice yield. (Personal communication)

S108. Y. Shao, X.W. Lu, W.Y. Cong, Influence of different ratio of nitrogen, phosphorus and potassium on yield and economic benefit of rice. *J. Shanghai Agric. Sci. Technol.* **3**, 41-42 (2007).

S109. Y.F. Wang, G.F. Mei, B. Ding, Q. Zhang, M.P. Zhu, Influence of different ration of nitrogen, phosphorus and potassium on yield and properties of rice. *Barley Cereal. Sci.* **4**, 46-48 (2007).

S110. H.X. Xiao, J.G. Li, Effects of formula fertilizer application with measuring soil nutrients test on rice yield in lower yielding paddy soil. *Guizhou Agric. Sci.* **35(6)**, 80-82 (2007).

S111. F.Y. Qian, G. Zhu, C.M. Zhu, Summary of ‘3414’ experiment of soil testing and fertilizer recommendation for rice in Huaiyin. *Mod. Agric. Sci. Technol.* **21,** 122 (2007).

S112. J.G. Li, Summary of experiment on accurate nitrogen fertilizer application in rice. *Mod. Agric. Sci. Technol.* **13**, 139-143 (2007).

S113. S.Q. Zhao, Q. Chen, D.W. Li, Discussion on response of fertilization to rice. *Mod. Agric. Sci. Technol.* **11,** 100-104 (2007).

S114. Y.Q. Shen, Research on effect of balance fertilization on rice. *Agric. Extension Services* **24(10)**, 34 (2007).

S115. Z.R. Liu, H.Z. Rao, Primary report on effect of slow/controlled release fertilizer application on rice. *Tillage Cult.* **3,** 29, 58 (2007).

S116. N.R. Lan, X.L. Zhang, C.X. Yang, H.D. Ling, L.B. Wang, The effect of different formulation application pattern on rice yield in Tongzi county. *Guizhou Agric. Sci.* **35(4)**, 66-68 (2007).

S117. G.Y. Sun, C.H. Gong, Z.Y. Wang, ‘3414’ fertilizer experiment for rice in Xiangshui county. *Anhui Agric. Sci. Bull.* **13(23)**, 116,134 (2007).

S118. S.Q. Li, J.D. Chen, Q. Zuo, Influences of optimizing fertilization on the growth and yield of rice-Wandao 68. *J. Anhui Agric. Sci.* **35(27)**, 8571-8573 (2007).

S119.Y.G. Xue, L.Z. Hou, J.Q. Zheng, J.X. Xiong, Study on “3414” experiment and its statistics analysis – a case of effect of different rate and ratio of nitrogen, phosphorus and potassium fertilizer application on rice yield in oil sand. The proceeding of arable land conversation quality in Jiangsu 241-248 (2008).

S120. Q.L. Shi, S.C. Teng, Z.W. Long, J.Z. Shi, J. Liu, Effects of different nitrogen, phosphorus and potassium amounts on rice yield. *Guizhou Agric. Sci.* **36(4)**, 43-45 (2008).

S121.C.X. Yang, J. Tan, Y.Q. Du, F.H. Cui, Effects of nitrogen, phosphorus and potassium

fertilizers on rice in calcareous purple paddy fields. *J. Chin. W. Normal. Univ. (Natural*

*Sciences)* **29(2)**, 148-152 (2008).

S122. Y.Y. Zhang, "3414" fertilizer experiment for rice in Jixi county. *Anhui Agric. Sci. Bull.* **14(20),** 51, 68 (2008).

S123. D.P. Tian, J. Zhang, Rice formula fertilization by soil test in percogenic purple mud field. *Mod Agric. Sci.* **15(7)**, 49-54 (2008).

S124. S.Y. Wang, Z.M. Kang, Experiment on balance fertilization parameters for rice. *Mod. Agric. Sci. technol.* **5**, 139 (2008).

S125. Z.Y. Wang et al., Rice formula fertilization by soil test in sandy area of Xiangshui county. *Mod. Agric. Sci. technol.* **(1)**, 111-113 (2008).

S126. G.Y. Sun, P.L. Feng, C.H. Gong, Rice formula fertilization with soil test in oil soil area of Xiangshui county. *Mod. Agric. Sci. technol.* **1**, 106-108 (2008).

S127. F. Li, L.M. Liu, T.M. Bo, Q.M. Zhao, Report on the study of “3414” experiments for hybrid middle-season rice. *Anhui Agricl. Sci. Bull.* **14(13)**, 79, 133 (2008).

S128.W.N. Wang, Y.Y. Wang, Z.Q. Yao, Study on “3414” fertilization experiment and fertilizer recommendation on Mid-season rice. *Hubei Agric. Sci.* **47(2)**, 1416-1419 (2008).

S129. Z.J. Yu, L.M. Tang, L.W. Chai, Study on “3414” fertilization experiment for rice in 2007 at Lai An county. *Mod. Agric. Sci. technol.* **6**, 133-134 (2008).

S130. H.L. Wang, X.W. He, “3414” fertilizer experiment in rice in 2008 at Jixi county, Anhui province. *Soils.* **41(2)**, 320-323 (2009).

S131. L.X. Wang, L. Li, J.P. Li, Application and effect of soil testing and fertilizer recommendation technique in paddy rice. *Chin. Agric. Sci. Bull.* **25(6)**, 155-158 (2009).

S132. X.Q. Wang, G.P. Zhang, T.Y. Lu, Effects of different combination of nitrogen, phosphorus and potassium on rice yields of Gangyou188 in the Alluvial Mud soil. *Guizhou Agric. Sci.* **37(1)**, 40-42 (2009).

S133. X.H. Liu, The effect of nitrogen, phosphorus and potassium fertilizer formula fertilization on yield of zhongyou 85 (a Hybrid rice variety). *Guizhou Agric. Sci.* **37(3)**, 39-41 (2009).

S134. K. Wang, Z.H. Bai, G.H. Mo, Influence of different ratio of nitrogen, phosphorus and potassium on yield of rice in yellow paddy. *Agric. Extension Services* **26(3)**, 63-65 (2009).

S135. R.C. Wu, H.C. Bao, Y.Q. Wu, H.F. Zhang, Effect of three factors with four levels of nitrogen, phosphorus and potassium fertilizer application for rice. *Agric. Equipment Technol.* **35(2)**, 38-40 (2009).

S136. J.H. Cheng, C.E. Zhang, L. Zheng, Studying the impacts of fertilization on rice by ‘3414’ experiment in Guannan, Jiangsu Province. *J. Hebei Agric.Sci.* **13 (3)**, 56-59 (2009).

S137. D.F. Kang, H.J. Jia, Y.F. Wang, C.P. Zeng, H.A. Li, Studying the impacts of fertilization on rice by ‘3414’ experiment in Houba zhen of Kaixian County. *J. Mod. Agric. Sci. Technol.* **3,** 160-162 (2009).

S138. H.J. Qian, The summary effects of “3414” incomplete experiment on rice. *J.S. China. Normal. Univ. (Natural Science Edition)* **1,** 105-110 (2009).

S139. Z.F. Wu, Influences of different nitrogen fertilization application pattern on rice yield. *Agric.Extension Services* **24(4)**, 41 (2007).

S140. X.L.Fang, Primary study on the rice performance with "control released fertilizer" application in demonstration experiment in Tongcheng. *Anhui Agric. Sci. Bull.* **13(24)**, 46,70 (2007).

S141. C.S. Fang, A.J. Hu, A study on response of fertilization to rice. *Mod. Agric. Sci. Technol.* **(13)**, 137-138 (2007).

S142. L.F. Zhu et al. Effects of application of ecological fertilizer combined with chemical fertilizer on rice yield and nitrogen use efficiency. *Chin. J. Rice. Sci.* **21(6)**, 631-636 (2007).

S143. T.J. Liao, J.Y. Zhao, Y. Huang, Z.H. Rao, Influence of potassium –ammonium dihydrogen phosphate on nutritional status in paddy rice. *J. S. Agric. Univ.* **24(2)**, 165-168 (2002).

S144. X.Y. Ma, G.H. Rui, B.L. Ye, A.Q. Xu, Effect of ratio of nitrogen and potassium on rice yield. *J. Anhui Agric. Sci.* **30(3)**, 416, 439 (2002).

S145. G.X. Ding, X. Xu, Y. Qian, W.P. Zhao, Rice nitrogen fertilizer rate experiment. *J. Anhui Agric. Sci.* **34(1)**, 115, 159 (2006).

S146. L.H. Yang, G.M. Gu, Z.Y. Ni, Y.J. Pan, Application of optimizating nitrogen fertilization technology for rice. *J. Shanghai Agric. Sci. Technol.* **5,** 85 (2006).

S147. G.P. Zhang, M.Y. Yang, Z.Q. Huang, X.Q. Wang, Effects of different nitrogen, phosphorus and potassium amount and combination on agronomic characters and yield of Zhongyou 85, a hybrid rice variety. *Guizhou Agric. Sci.* **36(4),** 46-47 (2008).

S148.Y.N.Wen, K.Wang,The relationship between nitrogen, phosphorus and potassium fertilizer application amount and rice yield in Sandy and Muddy Soil. *Guizhou Agric. Sci.* **36(4)**, 54-55 (2008).

S149. J.F. Wang, X.G. Hu, G.C. Zhang, Analysis on "3414" fertilizer effect experiment for rice at Jianhu county. *Anhui Agric. Sci. Bull.* **15(3)**, 96-97 (2009).

S150. Annual report of major demonstration counties of soil test and fertilizer recommendation project for rice. 2006-2009.

S151. Z.L. Zhu, S.L. Zhang, B. Yin, X.Y. Yan, Historical comparisons on the response curves of rice yield-nitrogen application rate in Tai Lake Region. *Plant. Nutr. fert. Sci*. **16 (1)**, 1-5 (2010).

S152. H. Yu, J.K. Huang, R. Scott, B. Loren, Soil fertility changes of cultivated land in eastern China. *Geograph. Res.* **22**, 380-388 (2003).

S153. H.J. Liu, Z.M. Lu, D.L. Zhao, Study on soil nutrients changes in Huaxian County. *Soil Fert.* **6**, 30-33 (2003).

S154. Q.L. Fu, K.N. Wu, Q.L. Lv, J. Lu, Soil quality dynamics in suburb of Zhengzhou. *J. Hebei Agric. Sci.* **8**(3), 53-56 (2004).

S155. Y.L. Sun, Q. Liu, Soil fertilities and its relationship with maize productivity in the North China Plain. *Soils* **41**(2), 274-277 (2009).

S156. W.D. Ma, L.J. Zhang, X.T. Wei, Analysis on the status of soil nutrients in Zhenping county in 2007. *Mod. Agric. Sci. Technol.* **18**, 237-238 (2009).

S157. P.H. Yang, W.L. Wang, The change trend and countermeasures of soil nutrients in DunLiangTian. *Henan Agric.* **15**, 29 (2010).

S158. W. H. Zhang, The status and improving strategy of soil nutrients in Mengzhou city. *Henan Agric.* **4**, 31-32 (2009).

S159. X.P. Wu, Evaluation of soil nutrient status in Nanle county. *Henan Agric.* **7**, 55 (2010).

S160. Y.L. Wang, Y.L. Han, J.F. Tan, X. Zhang, Analyses on fertilizer application and soil nutrient conditions in the Nanyang Basin. *Henan Sci.* **27**(3), 309-311 (2009).

S161. Q.Y. He, The soil fertility status and fertilization countermeasures in Suiyang district of Shangqiu city. *Mod. Agric. Sci. Technol.* **18**, 233-235 (2009).

S162. X.R. Cheng, D.Y. Wei, X.G. Zhang, The status of soil nutrients and fertilizer technology countermeasures in Sheqi County. *Bull. Agric. Sci. Technol.* **9**, 101-102 (2010).

S163. M. Zhu, Z.H. Ma, Y.L. Ren, Q.Y. He, The status and evaluation of soil nutrients and fertilization countermeasures in Suiyang District. *Anhui Agric. Sci. Bull.* **15**(11), 106-107 (2009).

S164. J.X. Jia, L.S. Ren,The nutrient content change of top layer soil and counter measures in Xinan county. *Chin. Agric. Technol. Extension.* **26**(4), 38-40 (2010).

S165. M.S. Zhang *et al*., Status of soil nutrients in farm land and fertilizer strategy in Xinxiang. *J. Hebei Agric. Sci.* **14**(13), 19- 21(2010).

S166. X.P. Tong, L. Li, The status of soil nutrients and fertilization technology in Xiuwu county. *Jilin Agric.* **8**, 93 (2010).

S167. S.Q. Yang, W.L. Wang, G.H. He, L. Ren, Analysis of soil nutrient condition in North Plain of Henan Province. *Acta. Agric. Jiangxi* **22**(12), 72-74 (2010).

S168. H.L. Yan, The status of soil nutrients and fertilization countermeasures in Yanjin county. *Mod. Agric. Sci. Technol.* **4**, 280-281(2011).

S169. X.S. Song, L.P. Li, R.J. Chen, Analysis on the relationship between application rate of nutrient and crop yield in Xinxiang City, Henan. *Guizhou Agric. Sci.* **36**(3), 89-92 (2008).

S170. L.N. Li, Study on the status of soil nutrients and fertilization in Jiaozuo City. *J. Henan Agric. Sci.* **2**, 84-86 (2006).

S171. L.X. Pei, Research on soil fertility in different land use in Xinyang City. Master Dissertation, Huazhong Agricultural University (2007).

S172. J. Su, J. Cheng, Preliminary analyses on the results of soil nutrient test in Minquan county. *Henan Agric.* **4**, 19 (2011).

S173. A.N. Zhu, J.B. Zhang, J.S. Yang, L.Y. Zhou, X.L. Xin, Changes in soil organic matter content under intensive crop production in typical fluvo-aquic soils regions. *Chin. J. Soil. Sci.* **41**(3), 532-536 (2010).

S174. Y.J. Yang, J.S. Yang, The variation of soil organic matter content in the salinity region of Yucheng city in Shandong Province. *Chin. J. Soil. Sci.* **36**(5), 647-651 (2005).

S175. X.B. Zeng, L.Y. Bai, L.F. Li, S.M. Su, The status and changes of organic matter, nitrogen, phosphorus and potassium under different soil using styles of Shouguang of Shangdong Province. *Acta. Ecologica. Sinica.* **29**(7), 3737-3746 (2009).

S176. H.J. Li *et al*., Soil fertility change in Dezhou. *Chin. Agric. Sci. Bull.* **25**(13), 134-137 (2009).

S177. B.H. Zhang *et al.,* Study on changes in nutrients of soil surface in intensified agricultural regions. *Shandong Agric. Sci.* **9**, 67-69 (2009).

S178. J.R. Cao, J. Dong, Y.J. Liu, Z.L. Li, The temporal and spatial distribution of soil nutrients in cultivated horizon in Liaocheng City. *Hubei Agric. Sci.* **49**(6), 1340-1343 (2010).

S179. L. Zhen *et al*., The changes of nutrient balances driven by adjusting crop structure at county level during 25 years—A case study in Huirnin County. Shandong Province. *P1ant Nutr. Fertil. Sci.* **13**(2), 213-222 (2007).

S180. X.L. Tian, N.K. Li, F.X. Zhang, L.C. Ma, G.Q. Liu, Horizontal variation of soil nutrients in Jiaxiang county Shandong province in the last twenty years. *Soils* **37**(3), 341-343 (2005).

S181. S.Z. Lv *et al*., Soil nutrients and fertilizing countermeasures in Zhaoyuan City. *Shandong Agric. Sci.* **3**, 82-84 (2008).

S182. B.K. Lei, Q. Chen, M.S. Fan, F.S. Zhang, Y.D. Gan, Changes of soil carbon and nitrogen in Shouguang intensive vegetable production fields and their impacts on soil properties. *Plant Nutr. Fertil. Sci.* **14**(5), 914-922 (2008).

S183. X.J. Li, Z.Q. Hu, N. Liu, G.X. Zhao, S.F. Tian, Spatial-temporal evolution of soil fertility in Yellow River Delta---Case study in Kenli County. *Plant Nutr. Fertili. Sci.* **12**(6), 778-783 (2006).

S184. B. H. Zhang, J.P. Zhang, Q.X. Tang, A.M. Huang, J. Dong, Study on the effect of irrigation on fertility in top layer soil. *Yellow River* **30**(10), 67-68 (2008).

S185. Z.X. Chen, Y.F. Li, Z.J. Liu, The primary oriented monitoring report of soil fertility in Tianjin. *Tianjin Agric. For.* **3**, 1-3 (2001).

S186. H.D. Liu, X.P. Li , G.P. Zhao, L.H. Yin, Present situation and its changing law of soil fertility in cultivated land in Shanxi. *J. Shanxi Agric. Sci*. 38(1), 73-77 (2010).

S187. X.S. Li, Changes of organic matter contents in agricultural soils in Guangde County, Xuancheng City of South Anhui Province. *Soils.* **42** (6), 924-927 (2010).

S188. C.H. Zhou, J.P. Zhang, B.H. Zhang, Z.J.Wang, Z.T. Liu, Changes and sustainable usage in soil nutrients in cultivated horizon in the west of Shandong Province. *J. Liaocheng Univ. (Nat. Sci.)* **21**(1), 77-80 (2008).

S189. F.G. Hu, C.Y. Yao, X.M. Zhang, W. Tao, Dynamic changes in fertility of paddy in Shouxian County. *J. Anhui Agric. Sci.* **32**(1), 76-77 (2004). *Univ.* **34**(2), 106-109 (2003).

S190. H.S. Dai, B. Song, K. Qiu, Condition of soil nutrient in farmland and countermeasure in Changfeng County. *Anhui Agric.* **10**, 14-15 (2004).

S191. X.Z. Yin, T.C. Wang, Y.H. Xiong, Analysis of soil fertility and fertilizer recommendation in Juchao District, Chaohu City. *Anhui Agric. Sci. Bull*. **8**(1), 53-54 (2002).

S192. B. Song, Analysis of soil nutrient conditions and fertilizing strategy in HeFei city. *Anhui Agric. Sci. Bull.* **15**(21), 100-101(2009).

S193. Z.G. Ye, Research on soil nutrient evolution in Bo zhou City. Master Dissertation, Anhui Agricultural University (2007).

S194 X.W. Xu, Regional distribution and variation of soil organic carbon storage in arable soils at different scales. Ph. D. Dissertation, Nanjing Agricultural University (2008).

S195. W.Y. Bian, Y.Y. Dong, J.M. Zhou, Variation characteristics of nutrient status of four soil genus in Zhejiang Province. *Acta. Agric. Zhejiangensis* **21**(4), 354-357 (2009).

S196. S. Wei, S.R. Zhang, L.J. Deng, Y.H. Liu, P.F. Xiao, Temporal and spatial variation of soil organic matter contents in Pixian County, Sichuan. *Chin. J. Soil. Sci.* **35**(3), 261-263 (2004).

S197. Y.F. Hu, L.J. Deng, S.R. Zhang, Study of soil nutrient variation and sustainable utilization in Yucheng County. *J. Soil Water Conser.* **18**(6), 124-128 (2004).

S198. X.Z. Zhang, T.X. Li, J.X. Zhou, R.S. Zhang, Study on nutrient balance and the dynamics of soil nutrients in Zi-tong County. *J. Sichuan Agric. Univ.* **22**, 53-57 (2004).

S199. T.X. Li, G.R. Ma, X.Z. Zhang, C.Q. Wang, Y. He, Farmland nutrients balance and dynamics of soil nutrients on county level in purple hilly area of Qianwei County, Sichuan Province. *J. Soil Water Conser*. **17**(1), 37- 40 (2003).

S200. L. Yang, Y.F. Hu, Z.L. Xu, J.D. Xia, L.J. Deng, Temporal variation characteristics of soil nutrient in hilly region of Mid-Sichuan Basin: A case study in yuxing town of Zhongjiang County. Chin. *J. Soil. Sci.* **40**(5), 1057-1062 (2009).

S201. J.P. Yang *et al*., The status of soil nutrients of top layer in Quxian. *Mod. Agricul. Sci. Technol.* **9**, 173, 175 (2009).

S202. S.C. Peng, G. Chen, Analysis of change in main fertility index of the farmland in Xichang City from 1985 to 2006. *J. Anhui Agric. Sci.* **36**(35), 15590-15592 (2008).

S203. X.Z. Zhang, J.X. Zhou, T.X. Li, Y.G. Yang, R.S. Zhang, Study on nutrients change in farmland and sustainable development in central hilly area of Sichuan. *J. Soil Water Conser.* **18**(2), 84-87, 132 (2004).

S204. K. Zhui, D.S. Xiang, Nutrient change and manure management of cultivated soil in Guizhou. *Guizhou Agric. Sci*. **32**(6), 82-83 (2004).

S205. Q.X. Luo, Z.Z. Li, G.R. Liu, Situation of fertilizer application and change in fertilizer efficiency in Jiangxi Province. *Acta. Agric. Jiangxi* **16**(3), 48-54 (2004).

S206. K.A. Ren, H.A. Xiao, L. Li, H.C. Peng, Changes in soil organic matter, nitrogen, phosphorus and potassium contents in paddy soils in Dongting Lake area. *Res. Agricul. Modernization* **26**(2), 150-153 (2005).

S207. M.G. Jiang, X.Y. Bo, P.C. Hu, Z.C. Chen, Long-term located monitoring in fertility variation and fertilizing effects of cultivated land in Ningyuan County. *Hunan Agric. Sci.* **1**, 54-57 (2011).

S208. Alatengxihuri *et al*., Effect of different land use on soil nutrients in farmland. *Res. Agric. Modernization* **31**(4), 492-495 (2010).

S209. H.F. Xiong, Q.Z. Liao, S.Q. Zan, Nutrient situation and change of top soil in farmland in Ezhou City, Hubei Province. *Agric. Technol.* **27**(5), 51-54 (2007.

S210. D.L. Wang, T.C. Ai, X.G. Cheng, Study on the evolution of soil fertility during 1981 and 2008 in Jiangxia Area. *Horticulture Seed* **1**, 87-89 (2011).

S211. W.X. Huang, The status of soil nutrients in Binyang. *Guangxi Agric. Sci.* **1**, 37-39 (2003).

S212. J.Y. He, Study on the change of soil nutrients in farmland of Fangcheng District. *J. Guangxi Agric.* **25(1)**, 7-9 (2010).

S213. P.R. Kuang, F.J. Zhu, P.Q. Zou, Monitoring and evaluation of soil fertility in Baiyun District, Guangzhou City. *Guangdong Agric. Sci.* **3**, 30-32 (2004).

S214. L.Z. Zeng, H.W. Zeng, Z. Zeng, X. He, J.F. Luo, Analysis of the soil nutrient change in Xingning city. *Guangdong Agric. Sci.* **4**, 92-93, 105 (2009).

S215. Y.X. Huang, L.F. Guo, Variation of the main fertility characters of paddy soil in Longyan city. *Fujian J. Agric. Sci.* **17**(4), 234-237 (2002).

S216. F.C. Qiu, The status of soil nutrients in paddy soils in Guangze County. *Fujian Agric.* **5**, 14 (2004).

S217. Y. Zhou, L.L. Zhu, X.L. Chen, Y.N. Deng, D.M. Wang, The status of nutrients and fertilizer recommendation of paddy soil in Ansha town, Yongan City. *Jiangxi Agric. Sci. Technol.* **9**, 10-12 (2003).

S218. Y.C. Huang, Soil fertility changes of the yellow podzolic paddy field and fertilization measures. *J. Minxi Vocational. Tech. Colg.* **12**(3), 92-95 (2010).

S219. R.Y. Liu, Fertility changing trends and fertilization countermeasures of paddy soil in Wuping county. Fujian Sci.Technol. *Trop. Crops* **35**(4), 1-4 (2010).

S220. L.D. Wang, The status of soil nutrients and fertilization strategy in paddy in Nan’an City. *Fujian Agric.* **7**, 12 (2006).

S221. S.M. Huang, D.J. Bao, X.R. Huangpu, H.C. Zhang, in *Changes of Soil Fertility in China*（eds M.G. Xu, et al.）204-205 (Beijing, China science and technology press, 2006).

S222. X.G. Tong, S.M. Huang, M.G. Xu, C.A. Lu, W.J. Zhang, Effects of the different long-term fertilizations on fractions of organic carbon in fluvo-aquic soil. *Plant Nutr. Fert. Sci.* **15**(4), 831-836 (2009).

S223. Fertilization systems and land use sustainability. Zhao Bingqiang et al., Eds, Beijing, Science press, 2012, pp: 15-55; 173-

S224. X.P. Li, X.J. Shi, Effect of long-term imbalanced fertilization on purple soil fertility. *Plant. Nutr. Fert. Sci.* **13** (1), 27-32 (2007).

S225. X.J. Shi, Nutrient cycling in rice-upland crop rotation system, Ph. D. Dissertation, China Agricultural University, Beijing, China (2003).

S226. K.Y. Liu, C.X. Lu, M.F. Chen, G.Y. Yi, in *Changes of Soil Fertility in China* (eds M.G. Xu, et al.,) 63 (Beijing, China science and technology press, 2006).
