# Supplementary figures and images for: Plant-Based Assessment of Inherent Soil Productivity and Contributions to China’s Cereal Crop Yield Increase since 1980
Source: PLoS One. 2013 Sep 18;8(9):e74617. doi: 10.1371/journal.pone.0074617 (PMC3776784; doi:10.1371/journal.pone.0074617)

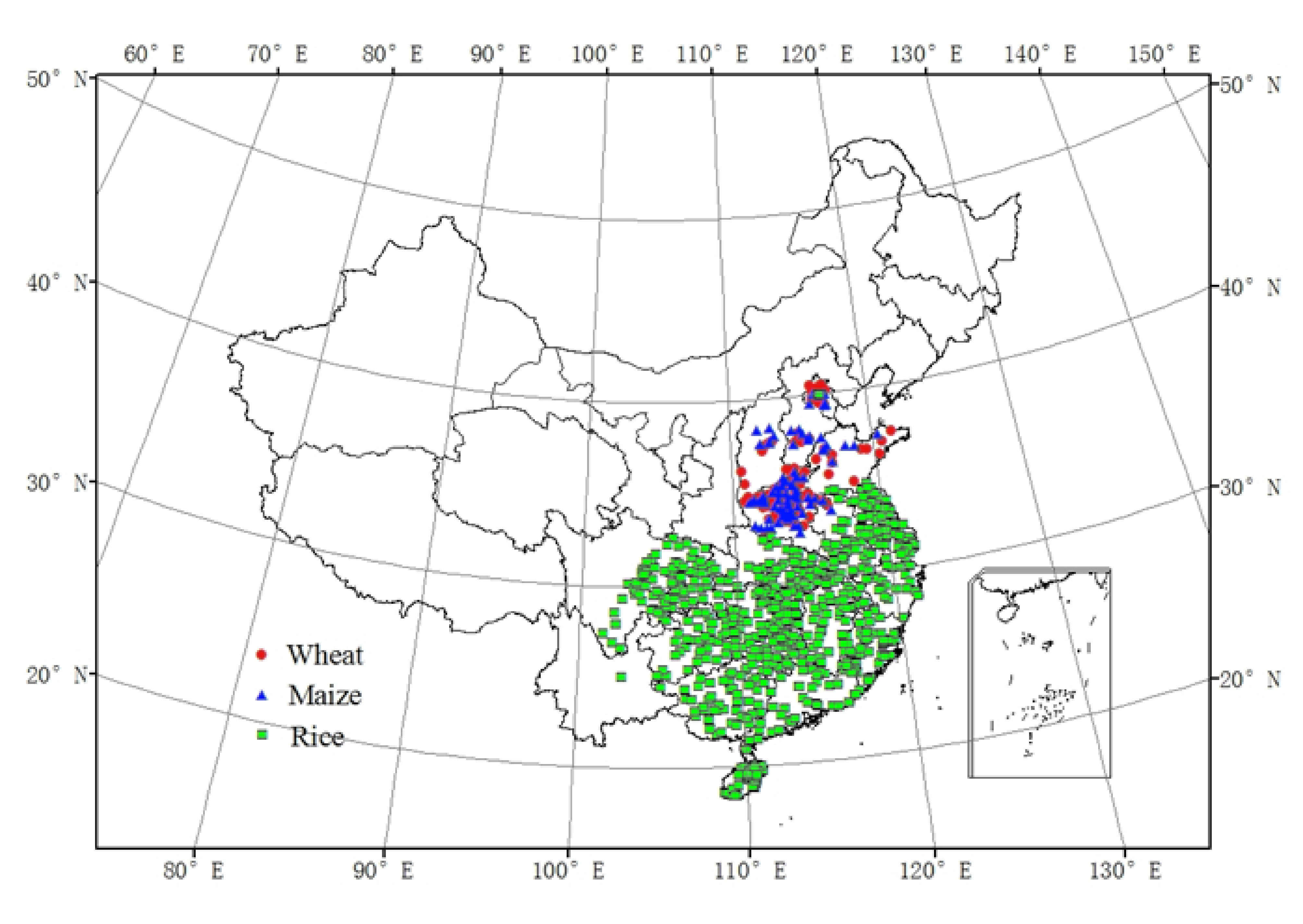

Supplement: Figure S1 — Geographical distribution of collected data. (TIF) [file pone.0074617.s002.tif]

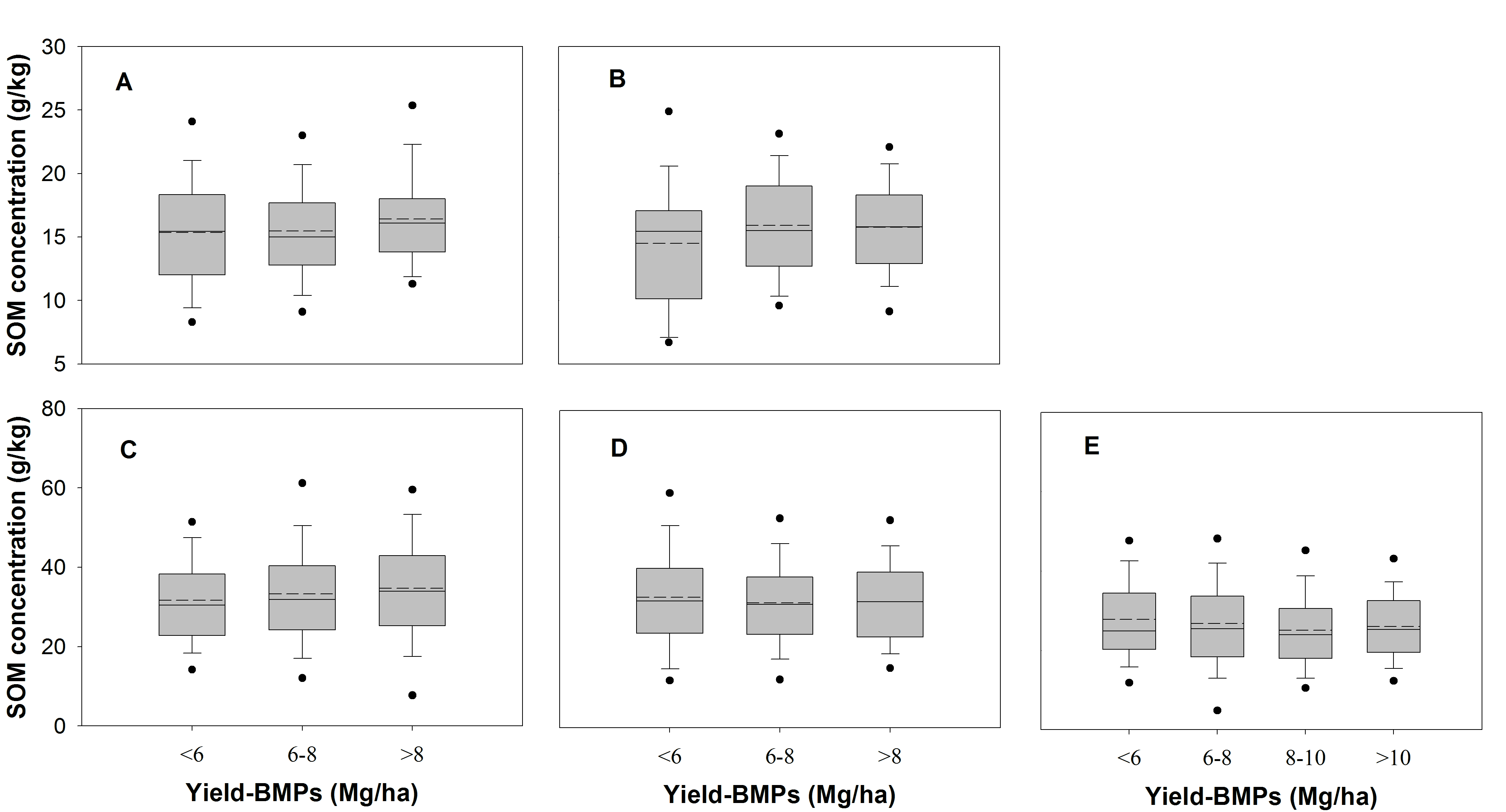

Supplement: Figure S2 — Relations between soil organic matter (SOM) concentrations and grain yield under best management practices on-farm trials (Yield-BMPs) in 5 major irrigated cereal-based cropping systems in China, references S1 to S151 in File S1. (a) winter wheat in north China (n=354); (b) summer maize in north China (n=425); (c) early rice in south of China (n=697); (d) late rice in south of China (n=688); (e) single rice in Yangtze River Basin (n=2474). Solid and dashed lines in this figure indicate median and mean of yield, respectively. The box boundaries indicate upper and lower quartiles, the whisker caps indicate 90th and 10th percentiles, and the circles indicate the 95th and 5th percentiles. (TIF) [file pone.0074617.s003.tif]

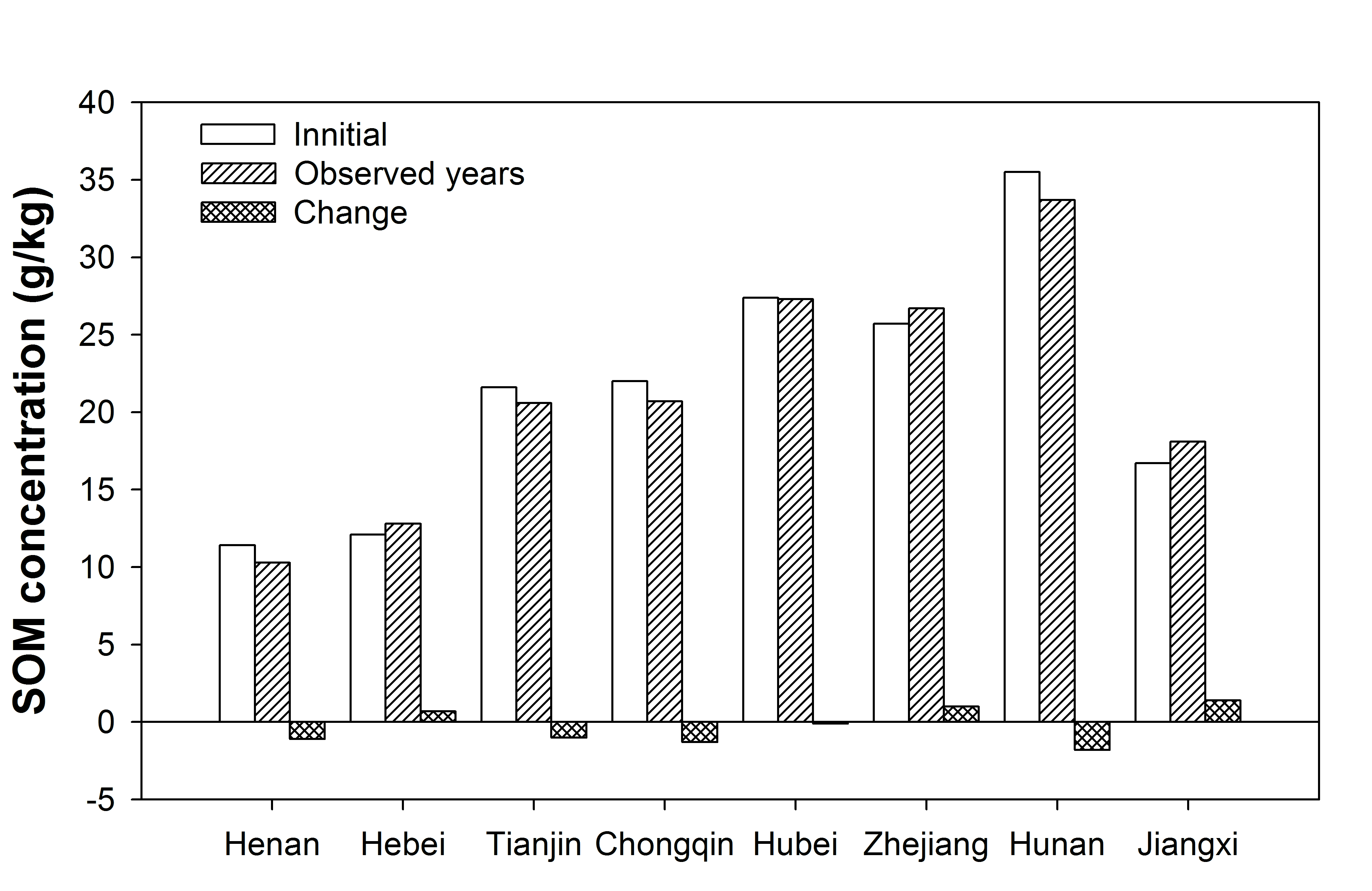

Supplement: Figure S3 — The changes in SOM concentration in control plots in long term experiments in major irrigated cereal based cropping systems, references S221 to S226 in File S1. (TIF) [file pone.0074617.s004.tif]
